# Genetic susceptibility to allergic bronchopulmonary aspergillosis in asthma: a genetic association study

**DOI:** 10.1186/s13223-016-0152-y

**Published:** 2016-09-27

**Authors:** Nicola L. D. Overton, David W. Denning, Paul Bowyer, Angela Simpson

**Affiliations:** 1 Manchester Fungal Infection Group (MFIG), The University of Manchester, Manchester, UK; 2 Division of Infection, Immunity and Respiratory Medicine, School of Biological Sciences, Faculty of Biology, Medicine and Health, Manchester Academic Health Science Centre, The University of Manchester and University Hospital of South Manchester NHS Foundation Trust, Manchester, UK

**Keywords:** ABPA, Asthma, Genetic susceptibility, TLR3, IL4R, IL13

## Abstract

**Background:**

In patients with asthma, the fungus *Aspergillus fumigatus* can cause allergic bronchopulmonary aspergillosis (ABPA). Familial ABPA is reported, and some genetic factors have been associated with the disease, however, these are small studies (n ≤ 38) and do not explain all cases of ABPA.

**Methods:**

We analysed SNPs in 95 ABPA patients, comparing frequencies to 152 atopic asthmatic and 279 healthy controls. Twenty two genes were selected from literature, and 195 tagging SNPs were analysed for genetic association with ABPA using logistic regression corrected for multiple testing. We also analysed monocyte-derived macrophage gene expression before and during co-culture with *A. fumigatus*.

**Results:**

Seventeen ABPA-associated SNPs (ABPA v Atopic asthma) were identified. Three remained significant after correction for multiple testing; IL13 rs20541, IL4R rs3024656, TLR3 rs1879026. We also identified minor differences in macrophage gene expression responses in the ABPA group compared to the control groups.

**Conclusions:**

Multiple SNPs are now associated with ABPA. Some are novel associations. These associations implicate cytokine pathways and receptors in the aberrant response to *A. fumigatus* and susceptibility to ABPA, providing insights into the pathogenesis of ABPA and/or its complications. We hope these results will lead to increased understanding and improved treatment and diagnostics for ABPA.

**Electronic supplementary material:**

The online version of this article (doi:10.1186/s13223-016-0152-y) contains supplementary material, which is available to authorized users.

## Background

The fungus *Aspergillus fumigatus* is ubiquitous and humans inhale several hundred conidia each day [1]. Most overtly immunocompetent individuals clear *A. fumigatus* conidia without infection or sequelae, however, some asthmatic patients develop allergic bronchopulmonary aspergillosis (ABPA) following inhalation and airway colonisation [1, 2]. The exact numbers affected is unclear, but if systemically sought, ABPA is identified in 1–8 % of asthmatics seen in hospital referral clinics [3, 4]. Presentation usually involves poorly controlled asthma, wheezing, expectoration of brown mucus plugs, and ‘pneumonia’. Elevated total blood IgE levels and IgE reactivity to *A. fumigatus* is observed in patients, and *A. fumigatus* is often isolated from sputum [1]. Often central bronchiectasis and mucoid impaction of bronchi with distal atelectasis occurs. Untreated ABPA can result in pulmonary fibrosis and eventually respiratory failure [1].

The human immune response to *A. fumigatus* involves many cell types, including macrophages and neutrophils. These phagocytose and kill the fungus and produce chemotactic and proinflammatory cytokines to continue and orchestrate the immune response. As macrophages are present in the airways they may be the first innate immune cell to contact inhaled fungi.

In ABPA patients, an allergic Th2 response develops on exposure to *A. fumigatus*. Stimulation of PBMCs with *Aspergillus* results in production of Th2 cytokines IL5 and IL13, and ABPA patients show increased *Aspergillus*-induced IL5 and IL13, and decreased IFNγ production, compared to healthy controls [5]. IgE production, eosinophil recruitment, and production of an abnormal host inflammatory response in the bronchi and bronchioles of the lungs is observed [6]. This is followed by excessive mucin production, eosinophil infiltration of the bronchial mucin, and development of the features of ABPA [1]. Why some asthmatic individuals develop ABPA while others are unaffected by exposure to *A. fumigatus* remains unclear, despite studies. In unsensitised mice, chronic intranasal administration of mould spores or extracts can lead to allergic lung inflammation, hyper-reactivity and lung remodelling [7], but the effect in humans is unclear. Cases of ABPA within families suggest a common genetic basis with low penetrance [8, 9]. In one case series from India, 5 % of cases were found to be familial in nature [10]. The structural gene CFTR has been previously associated with ABPA [11], and small genetic association studies (involving ≤38 patients) have identified associations between ABPA and SNPs in the immune genes IL4R, IL10, TLR9, SFTPA2 and HLA (HLA-DR) [12–16], however, these results do not explain all cases of ABPA, and the immune mechanisms that underlie ABPA remain unclear.

To advance our understanding of immune responses and genetic susceptibility to ABPA, we conducted a much larger genetic association study, involving almost 100 ABPA patients and using atopic asthmatic patients as controls, as well as a second control of healthy subjects. Candidate genes included known *A. fumigatus* recognition receptors (dectin-1, TLR2, TLR4 and TLR9 [17–20]) and other possible recognition receptors (TLR1, TLR3 and TLR6 [21, 22]), downstream response genes, many of which are upregulated in response to *A. fumigatus* (IL1α, IL1β, IL1RN, IL1RAP, IL6, IL10, IL17A, TNF-α, CCL2, TGFB1, PTX3) [20, 23], mannose binding lectin (MBL) and plasminogen, both of which bind *A. fumigatus* [23, 24], other Th1 (TNF-α, IL15) and Th2 (IL4, IL5, IL13, CCL17) cytokines [25, 26] and signal transducer and activator of transcription-3 (STAT3), which mediates IL-10 anti-inflammatory functions in macrophages and neutrophils [27]. Other immune genes not previously investigated in *Aspergillus* infection, such as DENN/MADD domain containing 1B (DENND1B), a negative regulator of the TNF-α receptor, and adenosine A2a receptor (ADORA2A) were also included [28]. In addition, we analysed expression of some of these immune genes in monocyte-derived macrophages (MDMs) at baseline and during co-culture with live *A. fumigatus,* comparing expression in cells derived from patients to those from control individuals.

## Methods

### Subjects

ABPA subjects, asthmatic controls and healthy controls were defined as described in Table 1. Both atopic (but fungally non-atopic) asthmatic and non-atopic asthmatic controls were recruited. ABPA subjects complicated by CCPA were excluded. Only Caucasian subjects were used. ABPA patients were recruited from the National Aspergillosis Centre [University Hospital of South Manchester (UHSM), UK] tertiary referral clinic from March 2006 to August 2010. Previous recruited healthy and asthmatic subjects were used as controls [29, 30]. The Local Research Ethics Committee approved the study and all participants gave informed consent. The healthy control subjects have been used as controls for previous studies into genetic susceptibility to CCPA [31, 32].

**Table 1:** Diagnostic criteria for recruited subjects

Disease	Diagnostic criteria
ABPA	*All the following are required* Total serum IgE 1000 IU/ml (at any time) Either positive SPT for *Aspergillus* or *Aspergillus* specific IgE Current or historical evidence of eosinophilia *Further indicators* Almost all patients have asthma (or cystic fibrosis, n = 3) and over 50 % central bronchiectasis on CT, but these were not required for inclusion Either recurrent obstruction (mucoid impaction) or episodes coughing up plugs of thick mucus (containing hyphae and eosinophils) Positive *Aspergillus* precipitins or raised *Aspergillus* IgG titre
Atopic (non-fungally atopic) asthmatic	*All the following are required* Physician diagnosed asthma No diagnosis of aspergillosis Negative SPT (at 3 mm cut-off) and/or IgE (<0.4) to all fungi tested, including *Alternaria alternata*, *Candida albicans*, *Cladosporium herbarum*, *Penicillium notatum, Trichophyton rubrum*, *A. fumigatus* Positive SPT (at 3 mm cut-off) and/or IgE (<0.4) to any allergen non-fungal allergen tested (e.g. mite, cat, dog and grasses) Note Only SPT or IgE need be completed, but if both are done and one is positive this is classed as a positive result
Non-atopic asthmatic	*All the following are required* Physician diagnosed asthma No diagnosis of aspergillosis Negative SPT (at 3 mm cut-off) and/or IgE (<0.4) to all allergens tested, including mite, cat, dog and grasses Negative SPT (at 3 mm cut-off) and/or IgE (<0.4) to all fungi tested, including *Alternaria alternata, Candida albicans, Cladosporium herbarum, Penicillium notatum, Trichophyton rubrum, A. fumigatus* Note: Only SPT or IgE need be completed, but if both are done and one is positive this is classed as a positive result
Healthy control	*All the following are required* No diagnosis of asthma No diagnosis of aspergillosis Negative SPT (at 3 mm cut-off) and/or IgE (<0.4) to all allergens tested, including mite, cat, dog and grasses Negative SPT (at 3 mm cut-off) and/or IgE (<0.4) to all fungi tested, including *Alternaria alternata, Candida albicans, Cladosporium herbarum, Penicillium notatum, Trichophyton rubrum, A. fumigatus*

### DNA and PBMC extraction from blood

For DNA, blood was collected in EDTA-treated blood collection tubes (Becton Dickinson; BD, Oxford, UK). This was centrifuged to separate the plasma and cellular sections and then DNA was then extracted from the cellular section using a phenol chloroform extraction method. Both the plasma and DNA were stored at −80 °C. For the previously recruited subjects, DNA had been collected previously [30].

For PBMC extraction, blood (≤80 ml) was collected in sodium heparin treated blood collection tubes (BD) then layered onto Ficoll-paque Plus (GE Lifesciences, Buckingham, UK) in a 1:1 ratio and centrifuged (23 °C, 0.5 h, 480*g*). The PBMC layer was transferred into growth media (RPMI 1640 with l-glutamine and NaHCO3, with 10 % heat inactivated FBS, 100 units/ml penicillin, and 0.1 mg/ml of streptomycin [Sigma-Aldrich Company Ltd, Dorset, UK]) and centrifuged to pellet the PBMCs (24 °C, 10 min, 390*g*). These PBMCs were frozen in freezing media [Heat inactivated FBS (Life Technologies Ltd, Paisley, UK) with 5 % DMSO Hybri-max (Sigma-Aldrich)] at a concentration of ~1 × 10^7^ cells/ml. Frozen PBMCs were transferred to liquid nitrogen for long term storage. Recovery and viability after freezing were assessed by trypan blue staining and found to be high (normally >90 %).

### Gene and SNP selection, genotyping, quality control and data analysis

Twenty two biologically plausible and previously associated candidate genes with immune functions were selected from the literature (Additional file 1: Table S1). These included genes involved in immune recognition and response, especially those involved in recognition and response to fungus specifically. It did not include the CFTR gene, which has structural functions.

A total of 253 haplotype tagging SNPs within these genes of interest were selected for genotyping, using the Genome Variation Server (GVS, http://gvs.gs.washington.edu/GVS/) (Additional file 1: Table S1). These were usually selected to encompass the entire gene, plus 2500 bp up- and 1500 bp down-stream.

Genotyping was completed on 237 SNPs using the Sequenom^®^ MassArray^®^ iPLEX™ Gold system. SNPs with Hardy–Weinberg Equilibrium p < 0.0001 or call rates <90 % were excluded from the analysis. After this, subjects with call rates <90 % were excluded from the analysis. Genotyping was completed in two rounds. In the first round, genotyping was completed successfully in 95 ABPA, 279 healthy and 152 atopic asthmatic subjects; an additional 14 atopic asthmatic subjects were genotyped successfully in the second round. Results were analysed using SNP and Variation Suite (SVS; version 7.4.3, Golden Helix). Redundant SNPs (r^2^ > 0.80) were excluded from analysis after evaluation of the LD within our population, as were SNPs that were monomorphic within our population. Statistical analysis was completed using Stata (Statacorp). Logistic regression was used to determine association for the remaining 195 SNPs using dominant and recessive models. Atopic asthmatics were used as controls for this analysis. Correction for multiple testing was completed using the Benjamini–Hochberg correction for False Discovery Rate [33]. A p value of p < 0.05 was considered significant. The p values were calculated using the R software [34]. The widely used Benjamini–Hochberg correction for False Discovery Rate is less stringent than the Bonferroni correction in which p values are multiplied by the number of comparisons. The Benjamini–Hochberg correction for FDR is shown to have higher power compared to other procedures including the Bonferroni method for multiple testing [35]. For SNPs associated with ABPA in the comparison to asthmatic subjects, a genetic association test was completed to identify the p value for the comparison to healthy subjects.

### Macrophage-*A. fumigatus *co-culture

Ten each of ABPA, non-atopic asthmatic and healthy subjects were selected for the macrophage-*A. fumigatus* co-culture experiment. MDMs were generated (approx. 2 × 10^5^/well), live *A. fumigatus* conidia added (4 × 10^5^/well) and RNA extracted as described previously [31]. Previous work shows that during the incubation period, proportions of the different fungal morphologies change, and that the time points of 0–3, 6 and 9 h are representative of exposure to conidia, germ tubes and hyphae respectively [31]. Experiments were repeated in triplicate.

### Measuring expression by MDMs

The human innate and adaptive immune responses RT2 profiler PCR array (SABiosciences) was used to measure expression of various genes related to those we genotyped in pooled RNA samples (1 μg) from each disease group (n = 10 subjects) using *HRPT1*, *RPL13A* and *GAPDH* as housekeeping normaliser genes. Arrays were repeated in triplicate. The investigated genes included TLRs (TLR1, 2, 3, 4, 9, 10) and related receptors (TREM1) and intracellular signalling genes (IRAK2, TRAF6, MYD88). For the majority of the arrays, the three housekeeping genes *HRPT1*, *RPL13A* and *GAPDH* were used as the normalizer genes, however, *HPRT1* was found to be variable in the healthy 9 h samples and so only *RPL13A* and *GAPDH* were used for the normalization of this experiment. Results were available for only two replicates of the asthmatic 6 h time point due to technical difficulties. The data was analysed using the manufacturer’s online data analysis tool (http://pcrdataanalysis.sabiosciences.com/pcr/arrayanalysis.php) to calculate fold changes relative to a calibrator.

### Statistical analysis

Statistical analysis was completed in Stata, SPSS (Version 16; SPSS Inc.) and GraphPad Prism (Version 5.02; GraphPad Software Inc.). Ages and % males were compared between the groups using Mann-Whitney tests as the data was not normally distributed. Expression data was analysed using T-tests and repeated measures one-way ANOVA, using data from the triplicate arrays. Standard deviation was calculated for these. Data for TLR1 was highly variable between replicates for the healthy group and this was excluded from analysis. As the one-way ANOVA required three replicates, the 6 h time point was excluded from this analysis of the asthma samples.

## Results

### Characteristics of study participants

The characteristics of the subjects recruited for genotyping are shown in Table 2. All are Caucasian. Those subjects with ABPA tended to be older than other groups (p < 0.0001), and had poorer lung function (% predicted FEV_1_, % predicted FVC or FEV_1_/FVC ratio, all p < 0.0001) (Table 2). Of the 97 ABPA, 280 healthy, 167 atopic asthmatic subjects recruited, 95 ABPA, 279 healthy and 166 atopic asthmatic subjects were successfully genotyped.

**Table 2:** Characteristics of patients and controls recruited

Characteristic	ABPA	Atopic asthma	Healthy
n	97	167	280
Age (year) (median, IQR)	61.7 year (54.0–69.5)	50.1 year (43.8–64.9)	47.0 year (44.2–50.6)
% Male	53.6 % (52/97)	41.3 %	40.4 % (113/280)
Asthma (%)	88.7 % (86/97)	100 % (167/167)	0 % (0/280)
Lung function tests (n)	67	165	255
FEV1 % predicted (median, IQR)	64.0 (51.0–78.0)	96.0 (78.4–108.2)	107.5 (99.6–117.2)
FVC % predicted (median, IQR)	96.0 (87.5–106.5)	108.9 (99.0–118.5)	114.7 (105.6–123.6)
FEV1/FVC ratio (median, IQR)	56.0 (47.9–64.0)	72.0 (63.0–79.0)	79.0 (75.0–83.0)
Markers of ABPA			
Bronciectasis (%)	67.0 % (65/97)	N/A	N/A
Total serum IgE (kU/L) (median, IQR) (n)	2250 (1100–4425) (96)	N/A	N/A
*Aspergillus* specific IgE (kUA/L) (median, IQR) (n)	9.7 (3.2–36.0) (62)	N/A	N/A

*IQR* interquartile range

The characteristics of the subjects selected for the gene expression study are shown in Table 3. The ABPA group were older than the healthy group (media age 59.5 years vs. 38.0 years, p = 0.0007), but not significantly different to the non-atopic asthmatic group (Table 3).

**Table 3:** Characteristics of patients and controls used for gene expression experiment

Characteristic	ABPA	Non-atopic asthma	Healthy
n	10	10	10
Age (year) (median, IQR)	59.5 year (55.7–66.1)	46.0 year (37.4–57.3)	38.0 year (31.2–51.1)
% Male	40.0 % (4/10)	20 % (2/10)	40 % (4/10)
Asthma (%)	70 % (7/10)	100 % (10/10)	0 % (0/10)
Lung function tests (n)	5	9	N/A
FEV1 % predicted (median, IQR)	75.0 (71.0–80.0)	85.0 (58.0–105.0)	N/A
FVC % predicted (median, IQR)	103.0 (102.0–106.0)	113.0 (89.0–116.0)	N/A
FEV1/FVC ratio (median, IQR)	60.8 (55.0–63.4)	78.0 (46.0–81.0)	N/A
Markers of ABPA			N/A
Bronciectasis (%)	*80 % (8/10)*	*50 % (5/10)*	*N/A*
Total serum IgE (kU/L) (median, IQR) (n)	4100 (2650–7075) (10)	71.9 (63.4–110.0) (10)	N/A
*Aspergillus* specific IgE (kUA/L) (median, IQR) (n)	34.0 (2.9–60.3) (6)	N/A	N/A

The recruitment criteria specified that the asthma control patients had a negative specific IgE or skin test to aspergillus. Therefore *Aspergillus* specific IgE level was not measured on every patient but was negative when measured

*IQR* interquartile range

### Multiple SNPs are associated with ABPA

**Fig. 1:**
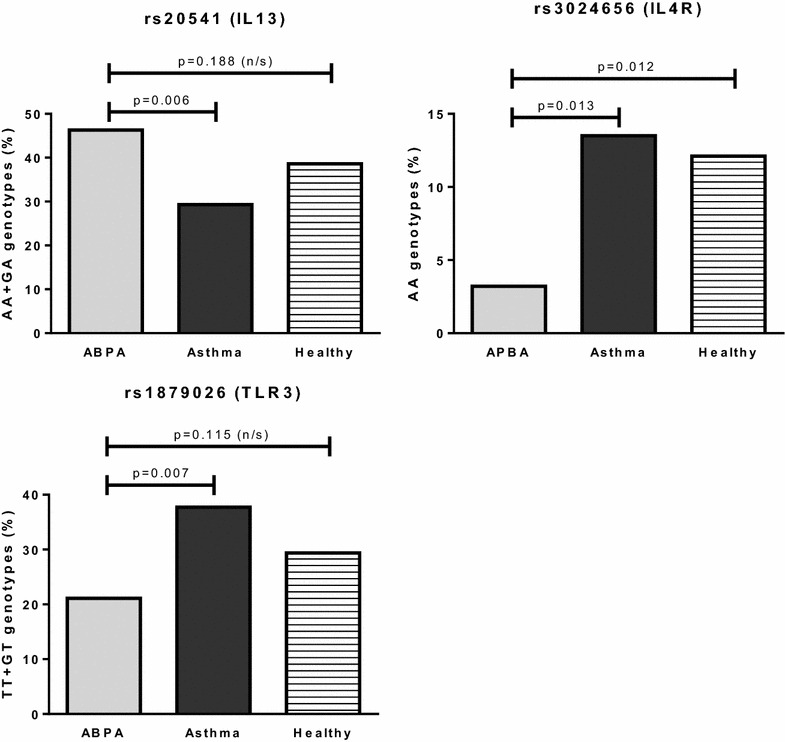
Genotype frequencies of SNPs associated with ABPA after correction for multiple testing

All the p values (uncorrected and FDR corrected) for all the models tested can be seen in Additional file 1: Table S2. Of the 195 SNPs analysed (Additional file 1: Table S1), 17 SNPs in nine genes were found to be associated with ABPA (p < 0.05, Table 4). Three of these survived correction for multiple testing (Table 4; Fig. 1). Compared to atopic asthmatics, ABPA patients were more likely to be carriers of the rare A allele of the exonic missense mutation in IL13 (rs20541). They were also more likely to carry the common G allele and common GG genotype of the intronic SNPs in IL4R rs3024656 and TLR3 rs1879026, respectively. Further analysis found that four of the SNPs associated with ABPA on the ABPA vs. Atopic asthma model were also associated on the ABPA vs. Healthy model (Additional file 1: Table S3). These were Dectin-1 rs7959451, IL13 rs1800925, IL4R rs3024656 and IL4R rs1029489.

**Table 4:** SNPs associated with ABPA

Gene	SNP	Alleles (M/m)	MAF (%)	Model for association	Genotype count				Odds ratio (95 % CI)	p value*	BH FDR p value*	Location
					Genotype	ABPA	Atopic asthma	Healthy^a^				
ADORA2A	rs2236624	C/**T**	26	CC + CT vs. TT	TT CC + CT	11 84	7 145	18 261	0.37 (0.14–0.99)	0.047	0.130	Intronic
DECTIN1	rs11053624	T/**C**	9	CC + TC vs. TT	TT CC + CT	72 23	132 20	233 46	2.11 (1.08–4.10)	0.028	0.086	5′ near gene
	rs7959451	C/**T**	14	TT + CT vs. CC	CC TT + CT	62 33	120 32	212 67	2.00 (1.12–3.55)	0.018	0.061	3′ UTR
IL13	rs20541	G/**A**	20	AA + GA vs. GG	GG AA + GA	51 44	116 48	167 105	2.08 (1.23–3.53)	0.006	*0.025*	Exonic (R > Q): missense
	rs1800925	C/**T**	18	TT + TC vs. CC	CC TT + TC	53 42	115 49	186 87	1.86 (1.10–3.14)	0.021	0.067	5′ near gene
IL17A	rs3819024	A/**G**	36	GG + GA vs. AA	AA GG + GA	33 62	74 78	115 164	1.78 (1.05–3.02)	0.032	0.097	5′ near gene
IL4R	rs3024656	**G**/A	33	GG + GA vs. AA	AA GG + GA	3 92	22 141	33 239	4.78 (1.39–16.44)	0.013	*0.045*	Intronic
	rs1029489	G/**A**	39	AA + GA vs. GG	GG AA + GA	24 71	64 100	113 160	2.00 (1.14–3.52)	0.016	0.054	3′ near gene
	rs6498012	G/C	38	GG + GC vs. CC	CC GG + GC	20 75	19 145	35 237	0.49 (0.25–0.98)	0.043	0.122	Intronic
MBL2	rs2099903	C/**A**	25	CC + CA vs. AA	AA CC + CA	11 84	6 146	21 258	0.31 (0.11–0.88)	0.027	0.086	3′ UTR
PLAT	rs8178880	**A**/G	4	GG + AG vs. AA	AA GG + AG	91 3	135 17	256 23	0.26 (0.07–0.92)	0.036	0.108	Intronic
PLG	rs4252053	A/**G**	15	GG + AG vs. AA	AA GG + AG	64 31	122 30	194 85	1.97 (1.10–3.54)	0.023	0.075	5′ near gene
TLR3	rs1879026	**G**/T	17	TT + GT vs. GG	GG TT + GT	75 20	94 57	197 82	0.44 (0.24–0.80)	0.007	*0.026*	Intronic
	rs10025405	A/**G**	43	GG + GA vs. AA	AA GG + GA	26 69	62 90	95 184	1.83 (1.05–3.18)	0.033	0.100	Intergenic (3′ of TLR3)
	rs5743303	A/**T**	19	TT + AT vs. AA	AA TT + AT	56 39	112 40	184 95	1.95 (1.13–3.36)	0.016	0.56	5′ near gene
	rs5743305	**T**/A	37	AA + TA vs. TT	TT AA + TA	48 47	54 98	117 162	0.54 (0.32–0.91)	0.020	0.67	5′ near gene
	rs7668666	C/**A**	26	AA + CA vs. CC	CC AA + CA	49 46	99 53	164 115	1.75 (1.04–2.96)	0.035	0.105	Intronic

Risk allele shown in bold. SNPs in bold remain significant after Benjamini–Hochberg adjustment for false discovery rate (FDR). Benjamini–Hochberg adjusted p value shown (BH FDR p value)

*CI* confidence interval; *M/m* Major allele/Minor allele

* p value calculated for the model indicated using logistic regression in Stata (ABPA v Atopic asthma)

^a^Genotype frequencies in our healthy population are shown for the readers interest but are not used for analysis

### Expression of TLR genes varies little over time after stimulation with *A. fumigatus*

**Fig. 2:**
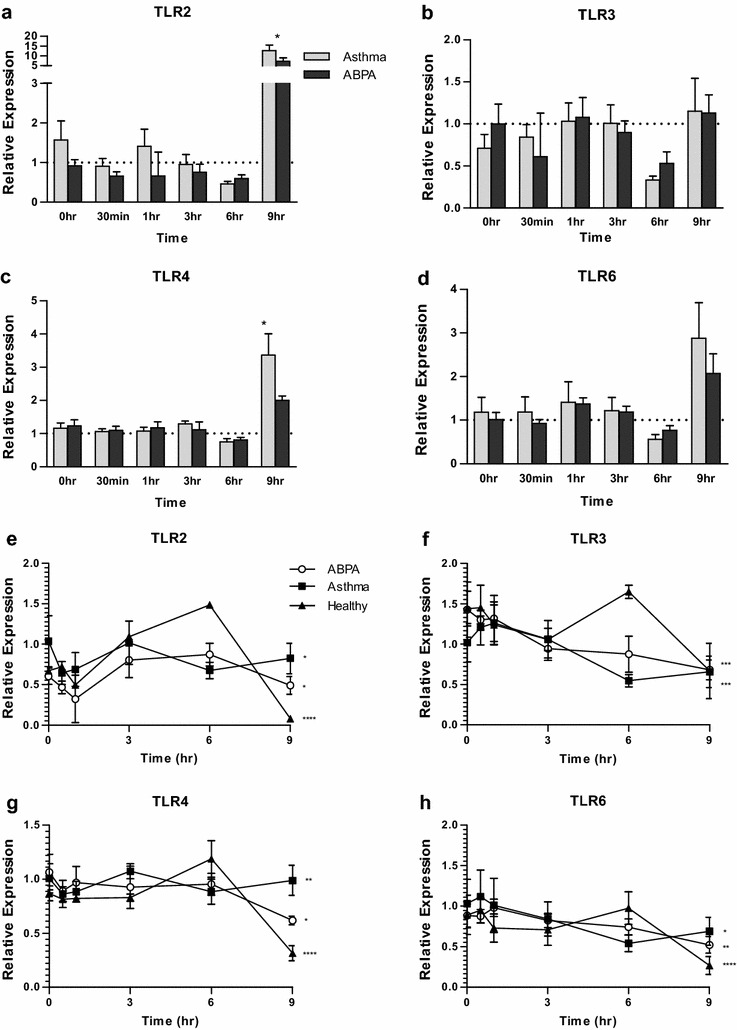
Expression of TLRs by MDMs from ABPA, asthmatic and healthy subjects (n = 10, pooled). **a**–**d** show expression in the ABPA (*black bars*) and asthma groups (*grey bars*) relative to the healthy group (*dotted line*) at each time point. *Stars* indicate significant differences between ABPA and asthma, calculated by t-test. **e**–**h** show expression in ABPA (*open circles*), asthmatic (*closed squares*) and healthy (*closed triangles*) subjects over time, relative to asthma 0 h. *Stars* indicate significant changes over time, calculated by repeated measures 1-way ANOVA. *p < 0.05; **p < 0.01; ***p < 0.001; ****p < 0.0001. *Bars* indicate standard deviation of the three replicates

**Fig. 3:**
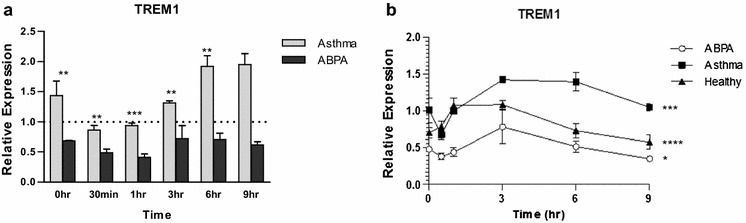
Expression of TREM1 by MDMs from ABPA, asthmatic and healthy subjects (n = 10, pooled). **a** shows expression in the ABPA (*black bars*) and asthma groups (*grey bars*) relative to the healthy group (*dotted line*) at each time point. *Stars* indicate significant differences between ABPA and asthma, calculated by t-test. **b** shows expression in ABPA (*open circles*), asthmatic (*closed squares*) and healthy (*closed triangles*) subjects over time, relative to asthma 0 h. *Stars* indicate significant changes over time, calculated by repeated measures 1-way ANOVA. *p < 0.05; **p < 0.01; ***p < 0.001; ****p < 0.0001. *Bars* indicate standard deviation of the three replicates

Expression of TLR2, TLR4, TLR6, and, to a lesser extent, TLR3, is lower in the ABPA and non-atopic asthmatic groups than in the healthy group at 6 h, and higher at 9 h (Fig. 2a–d). For TLR2 and TLR4 expression at 9 h is also significantly higher in asthma than in ABPA. The differences compared to the healthy group are a result of increased 6 h and reduced 9 h expression in the healthy subjects, which are not observed in the asthma or ABPA groups (Fig. 2e–h). The downstream signalling genes, TRAF6, IRAK2 and to a lesser extent MYD88 follow a similar expression pattern, although expression of other downstream signalling molecules does not (Additional file 1: Fig. S1). Expression of the activating receptor, triggering receptor expressed on myeloid cells 1 (TREM1) is significantly reduced in the ABPA group at baseline, compared to both the asthmatic and healthy groups, and remains lower at all time points (Fig. 3), which may suggest a reduced amplification of the TLR response in the ABPA group. Expression of further TLRs, including TLR9 and TLR10 is shown in the (Additional file 1: Fig. S2).

## Discussion

In the largest genetic association study of ABPA conducted to date, we have identified associations with SNPs in three immune genes, IL13 (rs20541), IL4R (rs3024656) and TLR3 (rs1879026), which remained significant after correction for multiple testing. Of these genes, only IL4R has previously been associated with ABPA; IL13 and TLR3 are novel candidate genes, which help to increase our understanding of the aberrant immune response occurring in ABPA patients. These associations now require replication in other populations to confirm their importance.

The association of SNPs in IL13 and IL4R with ABPA is interesting. IL13 and IL4 are Th2 cytokines and ABPA is known to involve a Th2 response [5]. PBMCs from ABPA patients show increased *Aspergillus*-induced IL-5 and IL-13 production, and decreased IFNγ production, compared to healthy controls [5]. Five SNPs in IL13 and IL4R were associated with ABPA before correction for multiple testing, and one in each remained significant after correction. One IL4R SNP has been previously associated with ABPA (rs1805010) [12]. This SNP has also been suggested to affect sensitivity to IL4 stimulation [12]. Unfortunately, this SNP failed the genotyping QC in the current study, however, a different SNP in IL4R (rs3024656) was found to be associated with ABPA. RS1805010 is a missense SNP in located in the 5′UTR of the IL4R gene (position 16:27344882), while rs3024656 is intronic (position 16:27358288). There is a very low LD between them (r^2^ = 0.16).

IL13 and IL4 act via the IL4R receptor and are involved in the allergic response to antigens such as those produced by *A. fumigatus* [5]. It has been shown that polymorphisms in IL4R and IL13 can have synergistic effects [36]. IL4 is a key cytokine required for differentiation of Th2 cells [26]. Uncontrolled Th2 are thought to be detrimental for both survival and disease progression in aspergillosis, and the identification of these SNPs supports this theory [17, 20, 37, 38].

The IL4R SNP (rs3024656) has been previously associated with only invasive squamous cell cervical cancer [39] and is not in high LD (r^2^ > 0.80) with any other SNPs, however, the IL13 SNP (rs20541) has been previously associated with many diseases and phenotypes, including asthma, atopy and serum IgE level [40–42]. In addition, rs20541 is in high LD with other SNPs (rs849, rs848, rs1295685, rs1295686) that have also been extensively associated with disease, including with asthma and serum IgE level [41]. As discussed, ABPA is an allergic disease often found in asthmatics, however, our use of atopic asthmatic controls should control for any association with asthma or non-fungal atopy in our population and suggests that this SNP is associated with ABPA or fungal atopy specifically. RS20541 is an exonic missense mutation in IL13, which results in an codon change from CGG to CAG, and an arginine (R) to glutamine (Q) substitution at amino acid position 110. Compared to atopic asthmatics, ABPA patients were more likely to be carriers of the rare A allele, coding for glutamine. This Q110 variant of IL13 appears to be less susceptible to clearance and more stable in plasma than the R110 variant, which may cause increased circulating levels in vivo [43]. This is supported by reports demonstrating significantly higher serum IL13 levels in human carriers of the Q110 homozygous genotype, compared to carriers of the R110 homozygous genotype [43]. As mentioned, *Aspergillus*-induced IL-13 is increased in PBMCs from ABPA patients compared to those from healthy controls [5]. The RS20541 A allele and the glutamine variant is also associated with elevated IgE levels [44]. IgE levels are also raised in ABPA [6]. In addition to rs20541, another SNP in IL13 (rs1800925) was found to be associated with ABPA, although this association did not survive correction for multiple testing. Like rs20541, IL13 rs1800925 has been extensively associated with atopic disease, including asthma, psoriatic arthritis and eczema [40, 42, 45, 46], as well as with allergy and IgE levels [42, 47]. It has also recently been associated with COPD [48]. The T allele of this SNP was associated with ABPA in the current study. This allele has been demonstrated to increase IL13 promoter activity in primary human and murine CD4 + Th2 cells by creation of a Yin-Yang binding site, which overlaps a STAT motif [49]. STAT6-mediated repression of IL13 transcription is reduced, allowing increased promoter activity and increased IL13 expression [49]. In addition, mitogen-activated PBMCs from humans homozygous for the T allele have been shown to secrete significantly higher levels of IL13 compared with those from CC and CT individuals [49]. The TT genotype is also associated with increased cord blood IgE [47]. The A allele (glutamine) of rs20541 and the T allele of rs1800925 may increase susceptibility to ABPA by increasing IL13 expression and increasing the allergic Th2 response, including IgE production, which is detrimental in aspergillosis. Other SNPs in IL13 and in other Th2 cytokines and receptors such as IL4 and IL4R could increase susceptibility to ABPA in a similar way.

Toll like receptors (TLRs) are known to be important in the recognition of *A. fumigatus* and as such it is likely that mutations in these receptors will influence susceptibility to aspergillosis. Traditionally, the most studied are TLR2, TLR4 and TLR9, however, recent work suggests that other TLRs such as TLR1, TLR3 and TLR6 may also be important [17–22]. Two SNPs in TLR1 (rs5743611, rs4833095) have been previously associated with IA, and one SNP in TLR9 (rs5743836) has been previously associated with ABPA [13, 22]. The study that identified the TLR9 SNP was small, involving only 22 ABPA patients and 88 controls [13]. None of these SNPs was found to be associated with ABPA in the current study; however, we did identify five SNPs at various locations within TLR3 as associated with ABPA. Only one of these (rs1879026) survived correction for multiple testing. This is an intronic SNP that has not been associated with disease in the past and is not in high LD (r^2^ > 0.80) with any other SNPs. TLR3 has only recently been implicated as a recognition receptor for *A. fumigatus*, with studies identifying a role in epithelial cell mediated protection against this fungus [19, 50], and the discovery of this association with ABPA is helpful in supporting this role.

We also analysed the expression of many of the TLR genes known to be expressed on human macrophages, in MDMs from healthy subjects, non-atopic asthmatics and ABPA subjects, in response to stimulation with *A. fumigatus*. As has been described by previous groups, we only observed small changes in gene expression over time after stimulation with *A. fumigatus* [19, 21, 23], however, 9 h TLR2 and TLR4 expression was significantly lower in the ABPA group compared to the asthmatic group. This could represent a differential response to the hyphal form of the fungus (predominant at the 9 h time point), or a differential time-dependant response to 9 h of culture. However, as the fold changes are low, it may also be that these differences and patterns are not biologically relevant. In addition, it may be that downstream signalling and the response to this is more important in the response to fungus than any change in TLR expression. This may account for the small changes in expression observed in the current study as well as in previous studies [19, 21, 23]. Alternatively, as the expression of some downstream signalling molecules follows a similar pattern to expression of the TLRs, there may indeed be a deficient response in the ABPA group.

The activating receptor TREM1 is found on neutrophils and monocytes and acts to amplifies inflammation induced by stimulation of TLRs, including TLR2 and TLR4 [51, 52]. Expression of TREM1 is reduced in the ABPA group, which may indicate a reduced ability to respond to TLR stimulation, including stimulation by fungi such as *A. fumigatus*. As TLR2 and TLR4 are key in the response to *A. fumigatus*, it could be that TREM1 plays a role in the response to *A. fumigatus* via interactions with these. We recently reported increased TREM1 expression by MDMs from CCPA subjects compared to those from healthy controls, and suggested that this might be an attempt to combat reduced baseline expression of TLRs by the CCPA group [32]. This does not appear to be the case in ABPA; in this situation, baseline TLR expression is not reduced, and increased TREM1 expression is not observed. Although both ABPA and CCPA occur in overtly immunocompetent subjects, they are very different diseases; CCPA is not considered an allergic disease and is not related to asthma. It is therefore not surprising that increased TREM1 expression is observed in MDMs from CCPA and reduced TREM1 expression is observed in ABPA. It is, however, interesting and suggests that this previously unstudied molecule may be important in susceptibility to fungal diseases and may benefit from further study.

We do not have information about the specific treatments patients were receiving at the time that blood was collected, however, because a small proportion of patients with ABPA in our clinic are prescribed corticosteroid therapy, either continuously or intermittently, it may be that some of those tested are currently taking this medication. The same is true for the asthmatic population. Systemic corticosteroid treatment can influence cell proliferation and RNA expression [53], however, as this effect of steroids on cells is temporary, we would expect any effect to be lost after the freezing, thawing and 15 days of culture that these cells undergo prior to use in our experiments.

While there remains uncertainty about the relevance of the expression profiles observed for the TLR genes, the association of the SNP in TLR3 with ABPA supports a role for this receptor in recognition of *A. fumigatus* and in response to this and TLR3 may affect susceptibility to ABPA, with SNPs that reduce the fungal recognition leaving people more susceptible to this disease.

We acknowledge that the alveolar macrophage could be considered as the ideal cell type in which to study interactions between host and pathogen as the initial interactions in the host are likely to occur within the lungs. However, if we had recruited only subjects that were fit for bronchoscopy we would have skewed the study population in favour of patients with mild ABPA. Collection of blood is a much less invasive procedure and is possible for subjects with all severities of ABPA; the use of MDMs therefore prevents population skewing.

We opted to use pooled samples for our gene expression work as although this prevents analysis of inter-patient variability, it allows for analysis of a greater number of genes (due to limited availability of patient cells). As has been discussed in detail previously [31], mean values for cases and controls are likely to be broadly similar to the results of pooled samples, however, we acknowledge the possibility that our pooled value could be skewed by a minority of individuals, Unfortunately, pooling also prevented us from associating gene expression with individual genotypes and therefore from identifying functional consequences of the SNPs. In addition, we felt that in the smaller gene expression study, the use of atopic asthmatic controls such as those used in the genetic association study, could present a risk as results could be skewed due to association with atopy to another non-fungal allergen (if two or three of the small control group were atopic to the same allergen), and so opted for non-atopic controls. In the genetic study, the larger control group with lots of different allergic profiles meant this was less likely to be a problem. We understand this is primarily a hypothesis generating study and further work will be required.

In addition, we acknowledge that the controls within our study are older than the cases, and this may mean that some controls may go on to develop ABPA later in life. This would reduce (rather than increase) our power to find associations with ABPA, and as such does not invalidate our findings. As a hypothesis generating study, and we appreciate that future work is required to support our results. This work will include replication of the genetic associations, functional work and experiments to confirm that gene expression differences translate to differences in protein levels. Despite this, we believe our results are important in suggesting novel genes and pathways that are associated with ABPA susceptibility and may be involved in the pathogenesis of ABPA. We hope these results are useful in providing novel directions for future research.

## Conclusions

The genetic association study presented here is the most extensive study into genetic susceptibility to ABPA to date, involving three times as many patients as most previous studies and investigating many more SNPs. We have identified SNPs in candidate genes including TLR3, IL4R and IL13 that are associated with ABPA, and which help to support the involvement of these previously theorised receptors and pathways in the immune response to *A. fumigatus* and in susceptibility to ABPA. We hope that this increased understanding will in future lead to developments in the treatment and diagnosis of ABPA.

## Additional file

10.1186/s13223-016-0152-y Supplementary tables and figures.

## Abbreviations

ABPA: allergic bronchopulmonary aspergillosis
SNP: single nucleotide polymorphism
IL: interleukin
TLR: toll like receptor
IgE: immunoglobulin E
Th: t helper
MDM: monocyte derived macrophage
UHSM: University Hospital of South Manchester
PBMCs: peripheral blood mononuclear cells
PCR: polymerase chain reaction
FDR: false discovery rate
