# Supplementary material for: Genetic susceptibility to allergic bronchopulmonary aspergillosis in asthma: a genetic association study
Source: Allergy Asthma Clin Immunol. 2016 Sep 27;12:47. doi: 10.1186/s13223-016-0152-y (PMC5037889; doi:10.1186/s13223-016-0152-y)
Supplement: Supplementary file 1 — 10.1186/s13223-016-0152-y Supplementary tables and figures. [file 13223_2016_152_MOESM1_ESM.doc]

**Genetic susceptibility to Allergic Bronchopulmonary Aspergillosis in asthma**

Nicola LD Overton^1,2^, David W Denning^1,2^, Paul Bowyer^1,2^, Angela Simpson^2*^

^1^Manchester Fungal Infection Group (MFIG), The University of Manchester, UK

^2^Faculty of Medical and Human Sciences, The University of Manchester; Manchester Academic Health Science Centre; University Hospital South Manchester NHS Foundation Trust; NIHR South Manchester Respiratory and Allergy Clinical Research Facility, Manchester, UK.

^*^**Corresponding author:** Prof Angela Simpson, University of Manchester, ERC Building, Second floor, Wythenshawe Hospital, Manchester M23 9LT, UK

Tel: +44 161 291 5871, Fax: +44 161 291 5730, Email: angela.simpson@manchester.ac.uk

Online Supplement

**Details of method of generating MDMs**

Frozen PBMCs were thawed, washed, and seeded onto 24 well plates at a concentration of 2x10^6^ per well. PBMCs were incubated at 37^o^C with 5% CO_2_ for 90 minutes in growth media (RPMI, penicillin-streptomycin, FBS), to allow plastic adherence of the monocytes, before media was replaced with growth media plus GM-CSF (PeproTech EC Ltd, London, UK) at 100ng/μL. Fifteen days of incubation followed, to allow maturation of the monocytes to monocyte derived macrophages (MDMs). Growth media (without GM-CSF) was replaced after the first three days of culture and as required thereafter. On day 15 the resulting MDM-enriched culture was used for the macrophage co-culture experiments. One plate of MDM-enriched cell cultures was generated for each timepoint (0hr, 30min, 1hr, 3hr, 6hr and 9hr). On day 15, RNA was extracted from the 0hr plate (unstimulated) and 4x10^5^ of live *A. fumigatus* conidia were added to the remaining plates. These were then incubated at 37^o^C with 5% CO_2_ until the required timepoint, when RNA was extracted.

The timepoints were chosen based on our analysis of fungal morphology over time, as well as the observations of Loeffler *et al* . both of which demonstrate that 3hr, 6hr and 9hr of culture results in exposure of MDMs to conidia, germtubes and hyphae respectively. The earlier timepoints (30min, 1hr) were also included to investigate early gene expression.

RNA was extracted using the RNeasy mini kit (Qiagen Ltd, Crawley, UK) according to the manufacturer’s instructions. Cells were lysed in the wells using buffer RLT and were homogenised using a QIAshredder (Qiagen). The optional on-column DNase treatment step was completed.

**Supplementary Table 1 - List of all SNPs selected for genotyping.**

| **Gene / SNP** | **Location** | **Genotyped** | **Alleles** | **MAF** | **Call rate** | **HWE**  **p-value** | **Analysed** |
| --- | --- | --- | --- | --- | --- | --- | --- |
| **ADORA2A** |  |  |  |  |  |  |  |
| rs7285057 | Upstream | ✓ | T/C | 10% | 0.99 | 0.768 | ✓ |
| rs2298383 | Intronic | ✓ | T/C | 43% | 1.00 | 0.788 | ✓ |
| rs8141793 | Intronic | ✓ | G/A | 4% | 1.00 | 0.708 | ✓ |
| rs11704811 | Intronic | ✓ | C/T | 9% | 1.00 | 0.655 | ✓ |
| rs2236624 | Intronic | ✓ | C/T | 26% | 1.00 | 0.340 | ✓ |
| **CCL2** |  |  |  |  |  |  |  |
| rs1024610 | Upstream | ✓ | A/T | 19% | 1.00 | 0.345 | ✓ |
| rs2857655 | 5’ near gene | ✓ | T/A | 5% | 1.00 | 0.842 | ✓ |
| rs2857656 | 5’ near gene | ✓ | G/C | 28% | 1.00 | 0.059 | ✓ |
| rs3760396 | 5’ near gene | ✓ | G/C | 23% | 1.00 | 0.145 | ✓ |
| rs3760399 | 5’ near gene | ✓ | A/G | 4% | 1.00 | 0.882 | ✓ |
| rs3917882 | Intergenic (5’) | No^1^ | - | - | - | - | - |
| rs4586 | Exonic (Cys/Cys) | ✓ | T/C | 38% | 1.00 | 0.099 | ✓ |
| **CCL17** |  |  |  |  |  |  |  |
| rs16956811 | Intronic | ✓ | T/G | 8% | 1.00 | 0.966 | ✓ |
| rs223827 | Intronic | ✓ | T/C | 39% | 1.00 | 0.927 | ✓ |
| rs223829 | 3’ near gene | ✓ | C/G | 23% | 1.00 | 0.814 | ✓ |
| rs223897 | Intronic | ✓ | C/T | 25% | 1.00 | 0.596 | No^# (rs223829)^ |
| rs4784805 | Intronic | ✓ | C/A | 3% | 1.00 | 0.460 | ✓ |
| rs497391 | 3’ near gene | ✓ | G/C^5 (G)^ | 0% | 0.00 | 1.000 | No^3, 5 (G)^ |
| rs886434 | Intronic | ✓ | C/T | 5% | 0.99 | 0.337 | ✓ |
| **DECTIN1 (CLEC7A)** | |  |  |  |  |  |  |
| rs10845046 | 3’ near gene | ✓ | A/G | 10% | 1.00 | 0.517 | No^# (rs16910526)^ |
| rs11053599 | Intronic | ✓ | C/A | 21% | 1.00 | 0.854 | ✓ |
| rs11053624 | 5’ near gene | ✓ | T/C | 9% | 1.00 | 0.377 | ✓ |
| rs11837849 | 5’ near gene | ✓ | T/G | 8% | 0.98 | 0.559 | ✓ |
| rs16910526 | 3’ UTR | ✓ | A/C | 9% | 0.97 | 0.666 | ✓ |
| rs3901532 | Intronic | ✓ | A/G | 25% | 1.00 | 0.152 | ✓ |
| rs6488262 | 3’ near gene | ✓ | A/G | 15% | 1.00 | 0.771 | ✓ |
| rs7309123 | Intronic | ✓ | C/G | 46% | 0.98 | 0.432 | ✓ |
| rs7311598 | Intronic | ✓ | A/G | 18% | 1.00 | 0.867 | ✓ |
| rs7959064 | 5’ near gene | ✓ | G/A | 45% | 0.97 | 0.640 | ✓ |
| rs7959451 | 3’ UTR | ✓ | C/T | 14% | 1.00 | 0.499 | ✓ |
| **DENND1B** |  |  |  |  |  |  |  |
| rs10922273 | Intronic | ✓ | C/T | 9% | 1.00 | 0.490 | ✓ |
| rs12141028 | Intronic | ✓ | G/A | 0% | 0.13 | 1.00 | No^3^ |
| rs12744898 | Intronic | ✓ | T/C | 21% | 1.00 | 0.984 | No^# (rs1747811)^ |
| rs12751508 | Intronic | ✓ | C/T | 13% | 1.00 | 0.880 | ✓ |
| rs1747811 | Intronic | ✓ | G/T | 21% | 1.00 | 0.854 | ✓ |
| rs17641481 | 3’UTR | ✓ | T/C | 4% | 0.99 | 0.535 | ✓ |
| rs1924518 | Intronic | ✓ | G/A | 20% | 0.98 | 0.832 | No^# (rs1747811)^ |
| rs2111930 | Intronic | ✓ | C/T | 46% | 1.00 | 0.593 | ✓ |
| rs2477063 | Intronic | ✓ | C/T | 20% | 1.00 | 0.870 | No^# (rs1747811)^ |
| rs2477073 | Intronic | ✓ | A/C | 34% | 0.80 | 0.157 | No^3^ |
| rs2477077 | Intronic | ✓ | C/T | 22% | 1.00 | 0.848 | ✓ |
| rs2488395 | Intronic | ✓ | C/T | 20% | 0.97 | 0.783 | No^# (rs1747811)^ |
| rs2488401 | Intronic | ✓ | C/T | 21% | 1.00 | 0.798 | No^# (rs2477077)^ |
| rs2786098 | Intronic | ✓ | C/A | 21% | 0.97 | 0.681 | ✓ |
| rs4282851 | Intronic | ✓ | G/A | 43% | 1.00 | 0.413 | ✓ |
| rs6661330 | Intronic | ✓ | T/A | 11% | 1.00 | 0.483 | ✓ |
| **IFNG** |  |  |  |  |  |  |  |
| rs1861494 | Intronic | ✓ | T/C | 29% | 0.99 | 0.322 | ✓ |
| rs2069716 | Intronic | ✓ | T/C | 5% | 0.99 | 0.407 | ✓ |
| rs2069718 | Intronic | ✓ | G/A | 39% | 0.99 | 0.471 | ✓ |
| rs2069727 | 3’ near gene | ✓ | T/C | 47% | 0.99 | 0.901 | ✓ |
| rs2069732 | 5’ near gene | No^1^ | - | - | - | - | - |
| rs2069707 | 5’ near gene | No^2^ | - | - | - | - | - |
| rs2069734 | 3’ UTR | ✓ | T/A^5 (T)^ | 0% | 0.62 | 1.000 | No^3, 5 (T)^ |
| rs2430561 | Intronic | No^1^ | - | - | - | - | - |
| **IL4R** |  |  |  |  |  |  |  |
| rs1029489 | 3’ near gene | ✓ | G/A | 39% | 0.99 | 0.813 | ✓ |
| rs1110470 | Intronic | ✓ | G/A | 48% | 0.99 | 0.513 | ✓ |
| rs1801275 | Exonic (Gln/Arg) | ✓ | A/G | 21% | 0.99 | 0.242 | ✓ |
| rs1805010 | Exonic (Ile/Leu) | ✓ | A/C/G | 0% | 0.50 | 0.000 | No^3, 4^ |
| rs1805011 | Exonic (Glu/Ala) | ✓ | A/C | 11% | 0.99 | 0.807 | ✓ |
| rs1805013 | Exonic (Ser/Leu) | ✓ | C/T | 5% | 0.99 | 0.591 | ✓ |
| rs1805015 | Exonic (Ser/Pro) | ✓ | C^5^ | 0% | 0.98 | 1.000 | No^5 (C)^ |
| rs2074570 | 3’ UTR | No^2^ | - | - | - | - | - |
| rs2074572 | Intronic | ✓ | C/T | 36% | 0.98 | 0.623 | ✓ |
| rs2234895 | Exonic (Asn/Asn) | ✓ | C/T | 9% | 0.99 | 0.199 | ✓ |
| rs2283563 | Intronic | ✓ | C/T | 30% | 0.99 | 0.510 | ✓ |
| rs2382720 | Intronic | ✓ | C/T | 47% | 0.98 | 0.185 | No^# (rs3024530)^ |
| rs3024530 | Intronic | ✓ | A/G | 47% | 0.98 | 0.756 | ✓ |
| rs3024536 | Intronic | ✓ | C/T | 14% | 0.99 | 0.685 | ✓ |
| rs3024585 | Intronic | ✓ | G/A | 43% | 0.99 | 0.225 | ✓ |
| rs3024610 | Intronic | ✓ | T/C | 49% | 0.98 | 0.490 | ✓ |
| rs3024622 | Intronic | ✓ | C/G | 34% | 0.99 | 0.922 | ✓ |
| rs3024647 | Intronic | ✓ | G/A^5 (G)^ | 0% | 0.00 | 1.000 | No^3, 5 (G)^ |
| rs3024656 | Intronic | ✓ | G/A | 33% | 0.99 | 0.458 | ✓ |
| rs3024658 | Intronic | ✓ | G/A | 5% | 0.99 | 0.151 | ✓ |
| rs4787948 | Intronic | ✓ | A/G | 31% | 0.99 | 0.766 | ✓ |
| rs6498012 | Intronic | ✓ | G/C | 38% | 0.99 | 0.983 | ✓ |
| rs8832 | 3’ UTR | ✓ | G/A | 44% | 0.99 | 0.815 | ✓ |
| **IL13** |  |  |  |  |  |  |  |
| rs1295687 | Intronic | ✓ | G/C | 6% | 0.99 | 0.286 | ✓ |
| rs1800925 | 5’ near gene | ✓ | C/T | 18% | 0.99 | 0.576 | ✓ |
| rs20541 | Exonic (Gln/Arg) | ✓ | G/A | 20% | 0.98 | 0.121 | ✓ |
| rs2066960 | Intronic | ✓ | C/A | 10% | 0.99 | 0.653 | ✓ |
| **IL15** |  |  |  |  |  |  |  |
| rs10519613 | 3’ UTR | ✓ | C/A | 11% | 0.99 | 0.320 | ✓ |
| rs10833 | 3’ UTR | ✓ | C/T | 37% | 0.99 | 0.864 | ✓ |
| rs12508866 | Intronic | ✓ | T/C | 23% | 0.99 | 0.531 | ✓ |
| rs1519551 | Intronic | ✓ | A/G | 46% | 0.99 | 0.483 | ✓ |
| rs1519553 | Intronic | ✓ | A/C | 29% | 0.99 | 0.743 | ✓ |
| rs17461269 | Intronic | ✓ | T/A | 32% | 0.99 | 0.157 | ✓ |
| rs17461627 | Intronic | ✓ | T/C | 1% | 0.99 | 0.708 | ✓ |
| rs17461899 | Intronic | ✓ | A/G | 10% | 0.99 | 0.353 | No^# (rs10519613)^ |
| rs2857261 | Intronic | ✓ | A/G | 44% | 0.98 | 0.709 | ✓ |
| rs3806798 | 5’ near gene | ✓ | T/A | 12% | 0.99 | 0.952 | ✓ |
| rs6842735 | Intronic | ✓ | G/T | 15% | 0.99 | 0.835 | ✓ |
| rs6850492 | Intronic | ✓ | G/A | 45% | 0.99 | 0.407 | ✓ |
| rs78795553 | Intronic | ✓ | T/A | 0% | 0.99 | 0.984 | ✓ |
| **IL17A** |  |  |  |  |  |  |  |
| rs12204016 | 3’ near gene | ✓ | C/T | 24% | 1.00 | 0.056 | No^# (rs1892279)^ |
| rs16882180 | 3’ near gene | ✓ | C/T | 36% | 1.00 | 0.057 | ✓ |
| rs17878503 | 3’ near gene | ✓ | G/T | 3% | 1.00 | 0.210 | ✓ |
| rs17879314 | 3’ near gene | ✓ | G/A | 18% | 0.97 | 0.934 | ✓ |
| rs17880818 | Intronic | ✓ | -/T | 0% | 0.00 | - | No^3^ |
| rs17884696 | Upstream | ✓ | C/T | 1% | 1.00 | 0.048 | ✓ |
| rs17884819 | Downstream | No^2^ | - | - | - | - | - |
| rs17886808 | Upstream | No^2^ | - | - | - | - | - |
| rs1892279 | 3’ near gene | ✓ | A/G | 24% | 1.00 | 0.055 | ✓ |
| rs1974226 | 3’ UTR | ✓ | G/A | 18% | 1.00 | 0.032 | ✓ |
| rs2275913 | Upstream | ✓ | G/A | 35% | 1.00 | 0.153 | ✓ |
| rs3748067 | 3’ UTR | ✓ | G/A | 9% | 1.00 | 0.690 | ✓ |
| rs3819024 | Upstream | ✓ | A/G | 36% | 1.00 | 0.072 | ✓ |
| rs3819025 | Intronic | ✓ | G/A | 5% | 1.00 | 0.178 | ✓ |
| rs4711998 | Upstream | ✓ | G/A | 25% | 0.98 | 0.620 | ✓ |
| rs7747909 | 3’ UTR | ✓ | G/A | 23% | 1.00 | 0.491 | ✓ |
| rs8193036 | Upstream | ✓ | T/C | 28% | 1.00 | 0.656 | ✓ |
| rs9395769 | 3’ near gene | ✓ | G/A | 13% | 1.00 | 0.000 | ✓ |
| **MBL2** |  |  |  |  |  |  |  |
| rs1031101* | 5’ near gene | ✓ | A/G | 14% | 1.00 | 0.292 | ✓ |
| rs10824792 | 3’ UTR | ✓ | T/C | 41% | 1.00 | 0.783 | ✓ |
| rs11003125 | 5’ near gene | ✓ | G/C | 36% | 1.00 | 0.044 | ✓ |
| rs11595876 | 3’ UTR | ✓ | T/C | 6% | 1.00 | 0.583 | ✓ |
| rs1800450* | Exonic (Gly/Asp) | No^1^ | - | - | - | - | - |
| rs1800451 | Exonic (Gly/Glu) | ✓ | G/A | 3% | 1.00 | 0.527 | ✓ |
| rs2099903 | 3’ UTR | ✓ | C/A | 25% | 1.00 | 0.184 | ✓ |
| rs3925313 | Integenic (5’) | ✓ | G/A | 22% | 1.00 | 0.138 | No^# (rs7096206)^ |
| rs4935047 | Intronic | ✓ | G/A | 45% | 0.97 | 0.370 | ✓ |
| rs5030737 | Exonic (Arg/Cys) | ✓ | C/T | 7% | 1.00 | 0.431 | ✓ |
| rs7095891* | 5’ near gene | No^2^ | - | - | - | - | - |
| rs7096206 | 5’ near gene | ✓ | C/G | 21% | 0.97 | 0.453 | ✓ |
| rs920724* | 5’ near gene | ✓ | A/G | 24% | 0.98 | 0.463 | ✓ |
| rs930509 | Intronic | ✓ | G/C | 17% | 0.97 | 0.726 | ✓ |
| **PLAT** |  |  |  |  |  |  |  |
| rs1136159 | Exonic (Ser/Ser) | ✓ | T/C | 13% | 1.00 | 0.692 | ✓ |
| rs2020920 | Intronic | ✓ | A/G | 6% | 1.00 | 0.106 | ✓ |
| rs2070712 | Intronic | ✓ | G/A | 27% | 0.99 | 0.713 | ✓ |
| rs2299609 | Intronic | ✓ | C/G | 38% | 1.00 | 0.104 | ✓ |
| rs4481580 | 3’ near gene | ✓ | G/A | 41% | 0.97 | 0.419 | ✓ |
| rs8178684 | Intronic | ✓ | C/T | 8% | 0.98 | 0.533 | ✓ |
| rs8178690 | Intronic | ✓ | C/T | 6% | 1.00 | 0.288 | ✓ |
| rs8178750 | Intronic | ✓ | C/T | 15% | 1.00 | 0.162 | ✓ |
| rs8178880 | Intronic | ✓ | A/G | 4% | 1.00 | 0.469 | ✓ |
| rs8178887 | Intronic | ✓ | G/A | 50% | 0.99 | 6x10^-183^ | No^4^ |
| rs8178890 | Intronic | ✓ | G/A | 6% | 1.00 | 0.416 | ✓ |
| rs879293 | Intronic | ✓ | G/A | 41% | 1.00 | 0.330 | ✓ |
| **PLG** |  |  |  |  |  |  |  |
| rs11060 | Exonic (Gly/Gly) | ✓ | G/T | 49% | 1.00 | 0.043 | ✓ |
| rs1317026 | Intronic | ✓ | C/T | 2% | 1.00 | 0.431 | ✓ |
| rs1652508 | Intronic | ✓ | C/T | 20% | 1.00 | 0.953 | No^# (rs783146)^ |
| rs1830521 | 5’ near gene | ✓ | A/C | 43% | 1.00 | 0.716 | ✓ |
| rs1835346 | Intronic | ✓ | T/C | 3% | 1.00 | 0.460 | ✓ |
| rs2144723 | 5’ near gene | ✓ | G/A | 49% | 1.00 | 0.162 | ✓ |
| rs4252050 | 5’ near gene | ✓ | A/G | 21% | 0.99 | 0.179 | ✓ |
| rs4252053 | 5’ near gene | ✓ | A/G | 15% | 1.00 | 0.392 | ✓ |
| rs4252055 | 5’ near gene | ✓ | G/A | 6% | 1.00 | 0.194 | ✓ |
| rs4252064 | Intronic | ✓ | G/A^5 (G)^ | 0% | 0.99 | 1.000 | No^5 (G)^ |
| rs4252069 | Intronic | No^1^ | - | - | - | - | - |
| rs4252075 | Intronic | No^1^ | - | - | - | - | - |
| rs4252087 | Intronic | ✓ | G/A | 31% | 0.98 | 0.287 | No^# (rs4252125)^ |
| rs4252099 | Intronic | ✓ | -/A | 30% | 1.00 | 0.288 | No^# (rs4252125)^ |
| rs4252108 | Intronic | ✓ | A/T | 31% | 1.00 | 0.693 | No^# (rs9295131)^ |
| rs4252125 | Exonic (Asp/Asn) | ✓ | G/A | 29% | 1.00 | 0.286 | ✓ |
| rs4252159 | Intronic | ✓ | G/A | 8% | 0.98 | 0.466 | ✓ |
| rs4252166 | Intronic | ✓ | G/C | 18% | 1.00 | 0.109 | ✓ |
| rs4252181 | 3’ UTR | ✓ | C/T | 28% | 1.00 | 0.210 | No^# (rs4252125)^ |
| rs4252185 | Intronic | ✓ | T/C | 10% | 0.99 | 0.975 | ✓ |
| rs4252192 | Intronic | ✓ | G/A | 4% | 1.00 | 0.619 | ✓ |
| rs4252200 | 3’ near gene | ✓ | A/G | 6% | 1.00 | 0.343 | ✓ |
| rs4709458 | Intronic | ✓ | T/C | 38% | 1.00 | 0.389 | ✓ |
| rs7454268 | Intronic | ✓ | C/T | 31% | 0.98 | 0.047 | ✓ |
| rs783146 | Intronic | ✓ | C/G | 17% | 1.00 | 0.556 | ✓ |
| rs783147 | Intronic | ✓ | C/T | 47% | 1.00 | 0.871 | ✓ |
| rs783167 | 3’ UTR | No^1^ | - | - | - | - | - |
| rs783176 | Intronic | ✓ | A/G | 19% | 1.00 | 0.187 | ✓ |
| rs783182 | Intronic | ✓ | C/T | 49% | 0.99 | 0.040 | No^# (rs11060)^ |
| rs9295131 | Intronic | ✓ | A/G | 33% | 1.00 | 0.734 | ✓ |
| rs9456577 | Intronic | ✓ | A/C | 3% | 0.99 | 0.702 | ✓ |
| rs9458011 | Intronic | ✓ | C/T | 7% | 0.99 | 0.776 | ✓ |
| **PTX3** |  |  |  |  |  |  |  |
| rs1840680 | Intronic | ✓ | G/A | 49% | 0.98 | 0.724 | ✓ |
| rs3845978 | Intronic | ✓ | C/T | 4% | 0.97 | 0.695 | ✓ |
| rs3911403 | Intronic | ✓ | T/A | 5% | 0.95 | 0.104 | ✓ |
| **STAT3** |  |  |  |  |  |  |  |
| rs1026916 | Intronic | ✓ | G/A | 37% | 0.99 | 0.067 | ✓ |
| rs12103893 | Intronic | ✓ | T/C | 18% | 0.95 | 0.127 | ✓ |
| rs17880347 | Intronic | ✓ | -/A | 7% | 0.97 | 0.988 | ✓ |
| rs17880900 | Intronic | ✓ | G/T | 0% | 0.99 | 0.000 | No^4^ |
| rs17881320 | Intronic | ✓ | C/A | 9% | 0.95 | 0.116 | No^# (rs17881940)^ |
| rs17881940 | Intronic | ✓ | G/C | 9% | 0.95 | 0.067 | ✓ |
| rs1905340 | Intronic | ✓ | C/A | 29% | 0.98 | 0.073 | ✓ |
| rs2293152 | Intronic | ✓ | G/C | 38% | 0.99 | 0.336 | ✓ |
| rs2306580 | Intronic | ✓ | C/G | 8% | 0.99 | 0.827 | ✓ |
| rs2306581 | Intronic | ✓ | C/A | 15% | 0.74 | 0.000 | No^3,4^ |
| rs6503695 | Intronic | ✓ | T/C | 35% | 0.95 | 0.124 | ✓ |
| rs6503697 | Intronic | ✓ | A/T | 29% | 0.95 | 0.049 | No^# (rs1905340)^ |
| rs7217655 | Intronic | ✓ | C/T | 37% | 0.99 | 0.164 | No^# (rs957971, rs1026916)^ |
| rs744166 | Intronic | ✓ | T/C | 44% | 0.97 | 0.133 | ✓ |
| rs8075442 | Intronic | ✓ | C/T | 0% | 0.99 | 0.000 | ✓ |
| rs8078731 | Intronic | ✓ | A/T | 18% | 0.98 | 0.233 | No^# (rs12103893)^ |
| rs957971 | Intronic | ✓ | C/G | 37% | 0.99 | 0.134 | No^# (rs1026916)^ |
| **TLR1** |  |  |  |  |  |  |  |
| rs4540055 | Intronic | ✓ | A/G/T | - | 0.98 | - | No^#^ ^(rs4833095)^ |
| rs4833095 | Exonic (Asn/Ser) | ✓ | T/C | 18% | 0.97 | 0.258 | ✓ |
| rs5743551 | 5’ near gene | No^2^ | - | - | - | - | - |
| rs5743565 | 5’ UTR | ✓ | A/G | 15% | 1.00 | 0.545 | No^# (rs4833095)^ |
| rs5743594 | Intronic | ✓ | C/T | 21% | 0.98 | 0.565 | ✓ |
| rs5743595 | Intronic | ✓ | T/C | 15% | 0.98 | 0.378 | ✓ |
| rs5743611 | Exonic (Arg/Thr) | ✓ | G/C | 8% | 1.00 | 0.709 | ✓ |
| rs5743618 | Exonic (Ser/Ile) | No^1^ | - | - | - | - | - |
| **TLR2** |  |  |  |  |  |  |  |
| rs1816702 | Intronic | ✓ | C/T | 11% | 1.00 | 0.566 | ✓ |
| rs1898830 | Intronic | ✓ | A/G | 33% | 0.99 | 0.296 | ✓ |
| rs3804099 | Exonic (Asn/Asn) | ✓ | T/C | 46% | 1.00 | 0.531 | ✓ |
| rs3804100 | Exonic (Ser/Ser) | ✓ | T/C | 9% | 1.00 | 0.378 | ✓ |
| rs4696480 | Intronic | ✓ | A/T | 48% | 1.00 | 0.466 | ✓ |
| rs5743704 | Exonic (Pro/His) | ✓ | C/A | 4% | 1.00 | 0.756 | ✓ |
| rs5743708 | Exonic (Arg/Gln) | ✓ | G/A | 3% | 1.00 | 0.345 | ✓ |
| rs7656411 | 3’ near gene | ✓ | T/G | 28% | 1.00 | 0.293 | ✓ |
| **TLR3** |  |  |  |  |  |  |  |
| rs10025405 | 3’ near gene | ✓ | A/G | 43% | 1.00 | 0.680 | ✓ |
| rs11721827 | Intronic | ✓ | A/C | 16% | 1.00 | 0.745 | ✓ |
| rs1879026 | Intronic | ✓ | G/T | 17% | 1.00 | 0.505 | ✓ |
| rs3775291 | Exonic (Leu/Phe) | ✓ | C/T | 30% | 1.00 | 0.986 | ✓ |
| rs3775292 | Intronic | ✓ | G/C | 21% | 1.00 | 0.771 | ✓ |
| rs5743303 | 5’ near gene | ✓ | A/T | 19% | 1.00 | 0.058 | ✓ |
| rs5743305 | 5’ near gene | ✓ | T/A | 37% | 1.00 | 0.664 | ✓ |
| rs5743313 | Intronic | ✓ | C/T | 21% | 0.99 | 0.371 | No^# (rs7657186)^ |
| rs5743314 | Intronic | ✓ | G/C | 21% | 1.00 | 0.386 | No^# (rs7657186)^ |
| rs6552950 | Intronic | ✓ | A/G | 25% | 0.94 | 0.078 | ✓ |
| rs7657186 | Intronic | ✓ | G/A | 21% | 1.00 | 0.669 | ✓ |
| rs7668666 | Intronic | ✓ | C/A | 26% | 1.00 | 0.003 | ✓ |
| **TLR4** |  |  |  |  |  |  |  |
| rs10759931 | Intergenic (5’) | ✓ | G/A | 37% | 0.99 | 0.897 | ✓ |
| rs11536857 | Intergenic (5’) | ✓ | C/T | 8% | 0.99 | 0.236 | ✓ |
| rs11536869 | Intronic | ✓ | A/G | 4% | 0.99 | 0.002 | ✓ |
| rs11536878 | Intronic | ✓ | C/A | 12% | 0.99 | 0.206 | ✓ |
| rs11536889 | 3’ UTR | ✓ | G/C | 15% | 0.97 | 0.456 | ✓ |
| rs11536891 | 3’ UTR | ✓ | T/C | 16% | 0.99 | 0.414 | ✓ |
| rs11536897 | 3’ near gene | ✓ | G/A | 5% | 0.99 | 0.032 | ✓ |
| rs12377632 | Intronic | ✓ | T/C | 36% | 0.98 | 0.978 | No^# (rs10759931)^ |
| rs1554973 | 3’ near gene | ✓ | T/C | 27% | 0.98 | 0.072 | ✓ |
| rs1927906 | 3’ near gene | ✓ | T/C | 11% | 0.99 | 0.602 | ✓ |
| rs1927907 | Intronic | ✓ | C/T | 14% | 0.98 | 0.355 | ✓ |
| rs2149356 | Intronic | ✓ | G/T | 34% | 0.98 | 0.697 | ✓ |
| rs4986791 | Exonic (Thr/Ile) | ✓ | C/T | 7% | 0.99 | 0.698 | ✓ |
| rs5030728 | Intronic | ✓ | G/A | 31% | 0.99 | 0.314 | ✓ |
| rs4986790 | Exonic (Asp/Gly) | No^1^ | - | - | - | - | - |
| **TLR9** |  |  |  |  |  |  |  |
| rs187084 | Upstream | ✓ | A/G | 40% | 1.00 | 0.714 | ✓ |
| rs352140 | Exonic (Pro/Pro) | ✓ | T/C | 46% | 1.00 | 0.388 | ✓ |
| rs5743836 | Upstream | ✓ | A/G | 15% | 1.00 | 0.826 | ✓ |
| **TLR10** |  |  |  |  |  |  |  |
| rs10856838 | Exonic (Ile/Ile) | ✓ | A/T | 12% | 1.00 | 0.652 | ✓ |
| rs11096955 | Exonic (Ile/Leu) | ✓ | T/G | 27% | 0.99 | 0.926 | ✓ |
| rs11466640 | Intronic | ✓ | G/A | 15% | 1.00 | 0.644 | ✓ |
| rs11466652 | Exonic (Lys/Lys) | ✓ | T/C | 11% | 1.00 | 0.634 | ✓ |
| rs11466657 | Exonic (Ile/Thr) | No^2^ | - | - | - | - | - |
| rs4129009 | Exonic (Ile/Val) | ✓ | T/C | 15% | 1.00 | 0.644 | ✓ |
| rs4513579 | Downstream | ✓ | T/C | 16% | 1.00 | 0.293 | No^# (rs4129009, rs7658893)^ |
| rs7658893 | Intronic | ✓ | G/A | 18% | 1.00 | 0.181 | ✓ |
| **TREM1** |  |  |  |  |  |  |  |
| rs12200981 | 3’ UTR | ✓ | A/G | 50% | 1.00 | 0.067 | ✓ |
| rs1351835 | Intronic | ✓ | A/T | 0% | 0.00 | - | No^3^ |
| rs2234237 | Exonic (Thr/Ser) | ✓ | T/A | 10% | 1.00 | 0.470 | ✓ |
| rs2234243 | Intronic | ✓ | C/T | 9% | 1.00 | 0.301 | ✓ |
| rs3789204 | 5’ near gene | ✓ | G/T | 28% | 1.00 | 0.420 | ✓ |
| rs3827632 | Intronic | ✓ | G/C | 11% | 1.00 | 0.214 | No^# (rs223423)^ |
| rs4711668 | Intronic | ✓ | C/T | 29% | 1.00 | 0.079 | ✓ |
| rs6939973 | Intronic | ✓ | A/G | 17% | 1.00 | 0.040 | ✓ |
| rs6940092 | 3’ UTR | ✓ | C/T | 40% | 1.00 | 0.028 | ✓ |

✓Yes. ^1^Failed primer design stage. ^2^Failed plexing stage. ^3^Failed SNP call rate, ^4^Failed HWE, ^5^Monomorphic in the current population (monomorphic allele in parenthesis), ^#^ In high LD with the SNP in parenthesis. * rs7095891 failed plexing and was replaced with rs920724, rs1800450 failed primer design and was replaced with rs1031101. MAF, minor allele frequency.

**Supplementary Table 2** - **All p-values for all SNPs. Results for both the dominant and recessive models are shown, with p-values (p), FDR corrected p-values (BH p), odds ratios (OR) and upper and lower confidence limits (UCL, LCL)**

| **Gene / SNP** | **Dominant model** | | | | | | | | | | | | | | | | | | | | | | | **Recessive model** | | | | |
| --- | --- | --- | --- | --- | --- | --- | --- | --- | --- | --- | --- | --- | --- | --- | --- | --- | --- | --- | --- | --- | --- | --- | --- | --- | --- | --- | --- | --- |
|  | **p** | **BH p** | | | | | | **OR** | | | | | | **LCL** | | | | | | **UCL** | | | | **p** | **BH p** | **OR** | **LCL** | **UCL** |
| **ADORA2A** |  |  | | | | | |  | | | | | |  | | | | | |  | | | |  |  |  |  |  |
| rs7285057 | 0.50 | 0.72 | | | | | | 0.79 | | | | | | 0.40 | | | | | | 1.56 | | | | 0.85 | 0.94 | 1.26 | 0.11 | 14.11 |
| rs2298383 | 0.46 | 0.69 | | | | | | 1.24 | | | | | | 0.70 | | | | | | 2.18 | | | | 0.09 | 0.22 | 0.57 | 0.29 | 1.09 |
| rs8141793 | 0.21 | 0.41 | | | | | | 0.54 | | | | | | 0.20 | | | | | | 1.41 | | | | N/D^1,2^ | | | | |
| rs11704811 | 0.83 | 0.93 | | | | | | 0.93 | | | | | | 0.47 | | | | | | 1.84 | | | | N/D^2^ | | | | |
| rs2236624 | 0.18 | 0.37 | | | | | | 1.43 | | | | | | 0.85 | | | | | | 2.39 | | | | 0.05 | 0.13 | 0.37 | 0.14 | 0.99 |
| **CCL2** |  |  | | | | | |  | | | | | |  | | | | | |  | | | |  |  |  |  |  |
| rs1024610 | 0.28 | 0.50 | | | | | | 1.34 | | | | | | 0.79 | | | | | | 2.27 | | | | 0.81 | 0.92 | 0.83 | 0.18 | 3.79 |
| rs2857655 | 0.61 | 0.79 | | | | | | 0.78 | | | | | | 0.30 | | | | | | 2.02 | | | | N/D^1^ | | | | |
| rs2857656 | 0.59 | 0.77 | | | | | | 0.87 | | | | | | 0.52 | | | | | | 1.45 | | | | 0.57 | 0.76 | 0.72 | 0.23 | 2.21 |
| rs3760396 | 0.87 | 0.95 | | | | | | 0.96 | | | | | | 0.57 | | | | | | 1.61 | | | | 0.21 | 0.42 | 3.86 | 0.46 | 32.60 |
| rs3760399 | 0.33 | 0.55 | | | | | | 0.59 | | | | | | 0.20 | | | | | | 1.71 | | | | N/D^1^ | | | | |
| rs3917882 | N/A | | | | | | | | | | | | | | | | | | | | | | | | | | | |
| rs4586 | 0.92 | 0.97 | | | | | | 0.97 | | | | | | 0.57 | | | | | | 1.66 | | | | 0.17 | 0.36 | 0.58 | 0.27 | 1.26 |
| **CCL17** |  |  | | | | | |  | | | | | |  | | | | | |  | | | |  |  |  |  |  |
| rs16956811 | 0.72 | 0.86 | | | | | | 0.88 | | | | | | 0.43 | | | | | | 1.79 | | | | N/D^1^ | | | | |
| rs223827 | 0.24 | 0.45 | | | | | | 0.72 | | | | | | 0.42 | | | | | | 1.24 | | | | 0.62 | 0.79 | 0.83 | 0.39 | 1.74 |
| rs223829 | 0.90 | 0.97 | | | | | | 1.03 | | | | | | 0.62 | | | | | | 1.73 | | | | 0.44 | 0.66 | 1.60 | 0.49 | 5.26 |
| rs223897 | N/A | | | | | | | | | | | | | | | | | | | | | | | | | | | |
| rs4784805 | 0.32 | 0.54 | | | | | | 0.45 | | | | | | 0.09 | | | | | | 2.19 | | | | N/D^1,2^ | | | | |
| rs497391 | N/A | | | | | | | | | | | | | | | | | | | | | | | | | | | |
| rs886434 | 0.50 | 0.72 | | | | | | 1.35 | | | | | | 0.56 | | | | | | 3.27 | | | | N/D^1,2^ | | | | |
| **DECTIN1 (CLEC7A)** |  |  | | | | | |  | | | | | |  | | | | | |  | | | |  |  |  |  |  |
| rs10845046 | N/A | | | | | | | | | | | | | | | | | | | | | | | | | | | |
| rs11053599 | 0.31 | 0.53 | | | | | | 0.75 | | | | | | 0.44 | | | | | | 1.30 | | | | 0.92 | 0.97 | 0.93 | 0.26 | 3.40 |
| rs11053624 | 0.03 | 0.09 | | | | | | 2.11 | | | | | | 1.08 | | | | | | 4.10 | | | | 0.74 | 0.87 | 0.62 | 0.04 | 10.07 |
| rs11837849 | 0.12 | 0.28 | | | | | | 1.70 | | | | | | 0.87 | | | | | | 3.30 | | | | 0.73 | 0.87 | 0.62 | 0.04 | 9.99 |
| rs16910526 | 0.49 | 0.71 | | | | | | 0.79 | | | | | | 0.41 | | | | | | 1.54 | | | | 0.20 | 0.40 | 0.22 | 0.02 | 2.17 |
| rs3901532 | 0.16 | 0.35 | | | | | | 1.45 | | | | | | 0.87 | | | | | | 2.42 | | | | 0.73 | 0.87 | 0.85 | 0.33 | 2.19 |
| rs6488262 | 0.47 | 0.70 | | | | | | 1.23 | | | | | | 0.70 | | | | | | 2.17 | | | | 0.56 | 0.76 | 0.62 | 0.12 | 3.12 |
| rs7309123 | 0.91 | 0.97 | | | | | | 1.03 | | | | | | 0.59 | | | | | | 1.81 | | | | 0.22 | 0.43 | 0.68 | 0.37 | 1.26 |
| rs7311598 | 0.42 | 0.64 | | | | | | 0.80 | | | | | | 0.46 | | | | | | 1.38 | | | | 0.24 | 0.45 | 2.58 | 0.54 | 12.43 |
| rs7959064 | 0.97 | 1.00 | | | | | | 1.01 | | | | | | 0.54 | | | | | | 1.90 | | | | 0.26 | 0.48 | 1.40 | 0.78 | 2.53 |
| rs7959451 | 0.02 | 0.06 | | | | | | 2.00 | | | | | | 1.12 | | | | | | 3.55 | | | | 0.64 | 0.80 | 0.62 | 0.09 | 4.48 |
| **DENND1B** |  |  | | | | | |  | | | | | |  | | | | | |  | | | |  |  |  |  |  |
| rs10922273 | 0.48 | 0.70 | | | | | | 1.26 | | | | | | 0.67 | | | | | | 2.37 | | | | 0.64 | 0.80 | 0.62 | 0.09 | 4.48 |
| rs12141028 | N/A | | | | | | | | | | | | | | | | | | | | | | | | | | | |
| rs12744898 | N/A | | | | | | | | | | | | | | | | | | | | | | | | | | | |
| rs12751508 | 0.53 | 0.73 | | | | | | 0.81 | | | | | | 0.42 | | | | | | 1.56 | | | | 0.85 | 0.94 | 1.26 | 0.11 | 14.11 |
| rs1747811 | 0.27 | 0.49 | | | | | | 1.34 | | | | | | 0.79 | | | | | | 2.28 | | | | 0.20 | 0.40 | 2.37 | 0.64 | 8.71 |
| rs17641481 | 0.26 | 0.47 | | | | | | 0.54 | | | | | | 0.19 | | | | | | 1.56 | | | | N/D^1^ | | | | |
| rs1924518 | N/A | | | | | | | | | | | | | | | | | | | | | | | | | | | |
| rs2111930 | 0.90 | 0.97 | | | | | | 0.97 | | | | | | 0.55 | | | | | | 1.69 | | | | 0.62 | 0.79 | 0.86 | 0.48 | 1.54 |
| rs2477063 | N/A | | | | | | | | | | | | | | | | | | | | | | | | | | | |
| rs2477073 | N/A | | | | | | | | | | | | | | | | | | | | | | | | | | | |
| rs2477077 | 0.20 | 0.40 | | | | | | 0.70 | | | | | | 0.40 | | | | | | 1.21 | | | | 0.18 | 0.37 | 2.93 | 0.62 | 13.85 |
| rs2488395 | N/A | | | | | | | | | | | | | | | | | | | | | | | | | | | |
| rs2488401 | N/A | | | | | | | | | | | | | | | | | | | | | | | | | | | |
| rs2786098 | 0.61 | 0.79 | | | | | | 1.15 | | | | | | 0.67 | | | | | | 1.96 | | | | 0.60 | 0.78 | 1.34 | 0.45 | 3.99 |
| rs4282851 | 0.28 | 0.49 | | | | | | 0.74 | | | | | | 0.44 | | | | | | 1.27 | | | | 0.66 | 0.82 | 1.16 | 0.60 | 2.24 |
| rs6661330 | 0.14 | 0.32 | | | | | | 1.63 | | | | | | 0.85 | | | | | | 3.11 | | | | 0.93 | 0.98 | 0.93 | 0.15 | 5.65 |
| **IFNG** |  |  | | | | | |  | | | | | |  | | | | | |  | | | |  |  |  |  |  |
| rs1861494 | 0.52 | 0.73 | | | | | | 0.85 | | | | | | 0.51 | | | | | | 1.40 | | | | 0.16 | 0.35 | 0.55 | 0.23 | 1.27 |
| rs2069716 | 0.17 | 0.37 | | | | | | 1.72 | | | | | | 0.79 | | | | | | 3.73 | | | | 0.70 | 0.85 | 0.58 | 0.04 | 9.33 |
| rs2069718 | 0.24 | 0.45 | | | | | | 1.39 | | | | | | 0.81 | | | | | | 2.41 | | | | 0.27 | 0.49 | 0.70 | 0.36 | 1.33 |
| rs2069727 | 0.57 | 0.76 | | | | | | 0.85 | | | | | | 0.48 | | | | | | 1.49 | | | | 0.11 | 0.25 | 1.74 | 0.89 | 3.42 |
| rs2069732 | N/A | | | | | | | | | | | | | | | | | | | | | | | | | | | |
| rs2069707 | N/A | | | | | | | | | | | | | | | | | | | | | | | | | | | |
| rs2069734 | N/A | | | | | | | | | | | | | | | | | | | | | | | | | | | |
| rs2430561 | N/A | | | | | | | | | | | | | | | | | | | | | | | | | | | |
| **IL4R** |  |  | | | | | |  | | | | | |  | | | | | |  | | | |  |  |  |  |  |
| rs1029489 | 0.02 | 0.05 | | | | | | 2.00 | | | | | | 1.14 | | | | | | 3.52 | | | | 0.37 | 0.58 | 0.73 | 0.37 | 1.44 |
| rs1110470 | 0.82 | 0.92 | | | | | | 0.94 | | | | | | 0.55 | | | | | | 1.61 | | | | 0.05 | 0.14 | 2.02 | 0.99 | 4.09 |
| rs1801275 | 0.45 | 0.67 | | | | | | 0.82 | | | | | | 0.49 | | | | | | 1.37 | | | | 0.18 | 0.38 | 4.19 | 0.51 | 34.60 |
| rs1805010 | N/A | | | | | | | | | | | | | | | | | | | | | | | | | | | |
| rs1805011 | 0.27 | 0.49 | | | | | | 0.70 | | | | | | 0.37 | | | | | | 1.32 | | | | 0.90 | 0.97 | 1.16 | 0.10 | 12.97 |
| rs1805013 | 0.47 | 0.70 | | | | | | 1.34 | | | | | | 0.60 | | | | | | 2.96 | | | | N/D^1^ | | | | |
| rs1805015 | N/A | | | | | | | | | | | | | | | | | | | | | | | | | | | |
| rs2074570 | N/A | | | | | | | | | | | | | | | | | | | | | | | | | | | |
| rs2074572 | 0.51 | 0.72 | | | | | | 1.20 | | | | | | 0.70 | | | | | | 2.03 | | | | 0.27 | 0.48 | 0.67 | 0.34 | 1.35 |
| rs2234895 | 0.65 | 0.81 | | | | | | 0.86 | | | | | | 0.46 | | | | | | 1.63 | | | | 0.31 | 0.53 | 0.29 | 0.03 | 3.19 |
| rs2283563 | 0.68 | 0.84 | | | | | | 1.11 | | | | | | 0.67 | | | | | | 1.85 | | | | 0.78 | 0.90 | 0.86 | 0.30 | 2.50 |
| rs2382720 | N/A | | | | | | | | | | | | | | | | | | | | | | | | | | | |
| rs3024530 | 0.26 | 0.48 | | | | | | 1.43 | | | | | | 0.76 | | | | | | 2.66 | | | | 0.73 | 0.87 | 0.90 | 0.51 | 1.61 |
| rs3024536 | 0.98 | 1.00 | | | | | | 1.00 | | | | | | 0.69 | | | | | | 1.46 | | | | 0.98 | 1.00 | 1.00 | 0.69 | 1.46 |
| rs3024585 | 0.70 | 0.85 | | | | | | 1.12 | | | | | | 0.63 | | | | | | 1.99 | | | | 0.06 | 0.17 | 0.54 | 0.28 | 1.04 |
| rs3024610 | 0.12 | 0.29 | | | | | | 1.66 | | | | | | 0.87 | | | | | | 3.15 | | | | 0.55 | 0.75 | 0.84 | 0.48 | 1.49 |
| rs3024622 | 0.33 | 0.55 | | | | | | 1.30 | | | | | | 0.77 | | | | | | 2.19 | | | | 0.10 | 0.24 | 0.53 | 0.25 | 1.12 |
| rs3024647 | N/A | | | | | | | | | | | | | | | | | | | | | | | | | | | |
| rs3024656 | 0.17 | 0.36 | | | | | | 0.70 | | | | | | 0.42 | | | | | | 1.16 | | | | 0.01 | 0.05 | 4.78 | 1.39 | 16.44 |
| rs3024658 | 0.76 | 0.88 | | | | | | 1.16 | | | | | | 0.46 | | | | | | 2.94 | | | | N/D^1,2^ | | | | |
| rs4787948 | 0.19 | 0.40 | | | | | | 1.41 | | | | | | 0.84 | | | | | | 2.37 | | | | 0.13 | 0.29 | 0.54 | 0.25 | 1.19 |
| rs6498012 | 0.24 | 0.45 | | | | | | 1.38 | | | | | | 0.81 | | | | | | 2.37 | | | | 0.04 | 0.12 | 0.49 | 0.25 | 0.98 |
| rs8832 | 0.08 | 0.20 | | | | | | 1.71 | | | | | | 0.94 | | | | | | 3.09 | | | | 0.81 | 0.92 | 0.93 | 0.50 | 1.72 |
| **IL13** |  |  | | | | | |  | | | | | |  | | | | | |  | | | |  |  |  |  |  |
| rs1295687 | 0.06 | 0.16 | | | | | | 2.02 | | | | | | 0.97 | | | | | | 4.21 | | | | N/D^2^ | | | | |
| rs1800925 | 0.02 | 0.07 | | | | | | 1.86 | | | | | | 1.10 | | | | | | 3.14 | | | | 0.62 | 0.80 | 0.72 | 0.19 | 2.73 |
| rs20541 | 0.01 | 0.02 | | | | | | 2.08 | | | | | | 1.23 | | | | | | 3.53 | | | | 0.15 | 0.33 | 0.28 | 0.05 | 1.56 |
| rs2066960 | 0.59 | 0.77 | | | | | | 0.84 | | | | | | 0.43 | | | | | | 1.62 | | | | 0.31 | 0.53 | 0.29 | 0.03 | 3.19 |
| **IL15** |  |  | | | | | |  | | | | | |  | | | | | |  | | | |  |  |  |  |  |
| rs10519613 | 0.54 | 0.74 | | | | | | 1.22 | | | | | | 0.65 | | | | | | 2.27 | | | | N/D^1^ | | | | |
| rs10833 | 0.90 | 0.97 | | | | | | 0.97 | | | | | | 0.58 | | | | | | 1.62 | | | | 0.74 | 0.87 | 0.89 | 0.43 | 1.83 |
| rs12508866 | 0.56 | 0.76 | | | | | | 0.86 | | | | | | 0.51 | | | | | | 1.43 | | | | 0.10 | 0.25 | 0.43 | 0.15 | 1.18 |
| rs1519551 | 0.57 | 0.76 | | | | | | 0.86 | | | | | | 0.50 | | | | | | 1.46 | | | | 0.25 | 0.47 | 0.71 | 0.39 | 1.28 |
| rs1519553 | 0.34 | 0.56 | | | | | | 1.28 | | | | | | 0.77 | | | | | | 2.12 | | | | 0.94 | 0.99 | 0.96 | 0.34 | 2.74 |
| rs17461269 | 0.16 | 0.34 | | | | | | 0.69 | | | | | | 0.42 | | | | | | 1.15 | | | | 0.92 | 0.97 | 0.96 | 0.45 | 2.06 |
| rs17461627 | 0.50 | 0.72 | | | | | | 1.75 | | | | | | 0.35 | | | | | | 8.85 | | | | N/D^1,2^ | | | | |
| rs17461899 | N/A | | | | | | | | | | | | | | | | | | | | | | | | | | | |
| rs2857261 | 0.91 | 0.97 | | | | | | 0.97 | | | | | | 0.56 | | | | | | 1.68 | | | | 0.63 | 0.80 | 1.17 | 0.63 | 2.17 |
| rs3806798 | 0.89 | 0.96 | | | | | | 1.05 | | | | | | 0.57 | | | | | | 1.93 | | | | 0.63 | 0.80 | 1.75 | 0.18 | 17.08 |
| rs6842735 | 0.50 | 0.72 | | | | | | 0.82 | | | | | | 0.46 | | | | | | 1.46 | | | | 0.86 | 0.95 | 1.16 | 0.21 | 6.47 |
| rs6850492 | 0.71 | 0.85 | | | | | | 1.11 | | | | | | 0.64 | | | | | | 1.93 | | | | 0.59 | 0.77 | 1.20 | 0.62 | 2.32 |
| rs78795553 | N/D^3^ | | | | | | | | | | | | | | | | | | | | | | | N/D^1,2^ | | | | |
| **IL17A** |  |  | | | | | |  | | | | | |  | | | | | |  | | | |  |  |  |  |  |
| rs12204016 | N/A | | | | | | | | | | | | | | | | | | | | | | | | | | | |
| rs16882180 | 0.17 | 0.37 | | | | | | 1.44 | | | | | | 0.85 | | | | | | 2.43 | | | | 0.41 | 0.63 | 0.74 | 0.36 | 1.51 |
| rs17878503 | 0.34 | 0.56 | | | | | | 0.56 | | | | | | 0.17 | | | | | | 1.82 | | | | N/D^1,2^ | | | | |
| rs17879314 | 0.44 | 0.66 | | | | | | 1.24 | | | | | | 0.72 | | | | | | 2.16 | | | | 0.11 | 0.26 | 0.16 | 0.02 | 1.50 |
| rs17880818 | N/A | | | | | | | | | | | | | | | | | | | | | | | | | | | |
| rs17884696 | 0.29 | 0.51 | | | | | | 0.31 | | | | | | 0.04 | | | | | | 2.72 | | | | N/D^1,2^ | | | | |
| rs17884819 | N/A | | | | | | | | | | | | | | | | | | | | | | | | | | | |
| rs17886808 | N/A | | | | | | | | | | | | | | | | | | | | | | | | | | | |
| rs1892279 | 0.73 | 0.87 | | | | | | 1.09 | | | | | | 0.65 | | | | | | 1.83 | | | 0.94 | | 0.99 | 0.97 | 0.40 | 2.34 |
| rs1974226 | 0.89 | 0.96 | | | | | | 1.04 | | | | | | 0.61 | | | | | | 1.79 | | | 0.72 | | 0.86 | 1.25 | 0.37 | 4.27 |
| rs2275913 | 0.25 | 0.47 | | | | | | 1.36 | | | | | | 0.81 | | | | | | 2.28 | | | 0.99 | | 1.00 | 1.00 | 0.46 | 2.16 |
| rs3748067 | 0.52 | 0.73 | | | | | | 1.25 | | | | | | 0.64 | | | | | | 2.44 | | | N/D^1,2^ | | | | | |
| rs3819024 | 0.03 | 0.10 | | | | | | 1.78 | | | | | | 1.05 | | | | | | 3.02 | | | 0.91 | | 0.97 | 0.96 | 0.45 | 2.02 |
| rs3819025 | 0.73 | 0.87 | | | | | | 1.16 | | | | | | 0.49 | | | | | | 2.73 | | | N/D^1,2^ | | | | | |
| rs4711998 | 0.68 | 0.84 | | | | | | 1.12 | | | | | | 0.66 | | | | | | 1.91 | | | 0.71 | | 0.85 | 0.79 | 0.24 | 2.68 |
| rs7747909 | 0.21 | 0.42 | | | | | | 1.40 | | | | | | 0.83 | | | | | | 2.36 | | | 0.71 | | 0.85 | 1.26 | 0.37 | 4.32 |
| rs8193036 | 0.28 | 0.49 | | | | | | 1.33 | | | | | | 0.80 | | | | | | 2.23 | | | 0.27 | | 0.49 | 0.60 | 0.24 | 1.50 |
| rs9395769 | 0.89 | 0.97 | | | | | | 1.04 | | | | | | 0.59 | | | | | | 1.84 | | | 0.71 | | 0.85 | 1.26 | 0.37 | 4.32 |
| **MBL2** |  |  | | | | | |  | | | | | |  | | | | | |  | | |  | |  |  |  |  |
| rs1031101* | 0.60 | 0.78 | | | | | | 0.86 | | | | | | 0.49 | | | | | | 1.51 | | | N/D^2^ | | | | | |
| rs10824792 | 0.56 | | 0.76 | | | | | | 0.85 | | | | | | 0.49 | | | | | | | 1.48 | 0.54 | | 0.74 | 0.82 | 0.43 | 1.56 |
| rs11003125 | 0.39 | | 0.61 | | | | | | 1.25 | | | | | | 0.75 | | | | | | | 2.10 | 0.13 | | 0.30 | 0.54 | 0.24 | 1.20 |
| rs11595876 | 0.11 | | 0.27 | | | | | | 2.04 | | | | | | 0.84 | | | | | | | 4.92 | N/D^2^ | | | | | |
| rs1800450* | N/A | | | | | | | | | | | | | | | | | | | | | | | | | | | |
| rs1800451 | 0.44 | | 0.66 | | | | | | 0.59 | | | | | | 0.15 | | | | | | | 2.27 | N/D^1,2^ | | | | | |
| rs2099903 | 0.73 | | | 0.87 | | | | | | 0.91 | | | | | 0.55 | | | | | | | 1.53 | 0.03 | | 0.09 | 0.31 | 0.11 | 0.88 |
| rs3925313 | N/A | | | | | | | | | | | | | | | | | | | | | | | | | | | |
| rs4935047 | 0.48 | | | 0.70 | | | | | | 0.81 | | | | | | 0.46 | | | | | | 1.45 | 0.27 | | 0.49 | 1.50 | 0.73 | 3.09 |
| rs5030737 | 0.38 | | | 0.60 | | | | | | 1.40 | | | | | | 0.67 | | | | | | 2.92 | N/D^2^ | | | | | |
| rs7095891* | N/A | | | | | | | | | | | | | | | | | | | | | | | | | | | |
| rs7096206 | 0.85 | | | 0.94 | | | | | | 1.05 | | | | | | 0.61 | | | | | | 1.81 | 0.97 | | 1.00 | 1.03 | 0.28 | 3.74 |
| rs920724* | 0.11 | | | 0.25 | | | | | | 0.64 | | | | | | 0.38 | | | | | | 1.10 | 0.50 | | 0.72 | 1.60 | 0.40 | 6.36 |
| rs930509 | 0.67 | | | 0.84 | | | | | | 0.88 | | | | | | 0.49 | | | | | | 1.58 | 0.21 | | 0.42 | 0.39 | 0.09 | 1.68 |
| **PLAT** |  | | |  | | | | | |  | | | | | |  | | | | | |  |  | |  |  |  |  |
| rs1136159 | 0.15 | | | | 0.33 | | | | | | 1.55 | | | | | | 0.85 | | | | | 2.84 | N/D^1^ | | | | | |
| rs2020920 | 0.48 | | | | 0.70 | | | | | | 1.37 | | | | | | 0.57 | | | | | 3.31 | N/D^1,2^ | | | | | |
| rs2070712 | 0.20 | | | | 0.41 | | | | | | 0.72 | | | | | | 0.43 | | | | | 1.20 | 0.08 | | 0.20 | 3.11 | 0.87 | 11.13 |
| rs2299609 | 0.56 | | | | 0.76 | | | | | | 0.86 | | | | | | 0.51 | | | | | 1.44 | 0.43 | | 0.65 | 1.36 | 0.63 | 2.94 |
| rs4481580 | 0.85 | | | | 0.94 | | | | | | 0.95 | | | | | | 0.55 | | | | | 1.63 | 0.29 | | 0.51 | 1.51 | 0.70 | 3.27 |
| rs8178684 | 0.70 | | | | 0.85 | | | | | | 1.15 | | | | | | 0.56 | | | | | 2.38 | N/D^1^ | | | | | |
| rs8178690 | 0.40 | | | | 0.62 | | | | | | 1.43 | | | | | | 0.63 | | | | | 3.23 | N/D^1,2^ | | | | | |
| rs8178750 | 0.72 | | | | 0.86 | | | | | | 1.11 | | | | | | 0.63 | | | | | 1.97 | N/D^1^ | | | | | |
| rs8178880 | 0.04 | | | | 0.11 | | | | | | 0.26 | | | | | | 0.07 | | | | | 0.92 | N/D^1^ | | | | | |
| rs8178887 | N/A | | | | | | | | | | | | | | | | | | | | | | | | | | | |
| rs8178890 | 0.29 | | | | 0.51 | | | | | | 1.56 | | | | | | 0.68 | | | | | 3.59 | N/D^1^ | | | | | |
| rs879293 | 0.46 | | | | 0.68 | | | | | | 1.23 | | | | | | 0.71 | | | | | 2.16 | 0.66 | | 0.82 | 1.16 | 0.59 | 2.28 |
| **PLG** |  | | | |  | | | | | |  | | | | | |  | | | | |  |  | |  |  |  |  |
| rs11060 | 0.60 | | | | 0.78 | | | | | | 1.17 | | | | | | 0.64 | | | | | 2.16 | 0.12 | | 0.27 | 0.62 | 0.34 | 1.13 |
| rs1317026 | 0.82 | | | | 0.92 | | | | | | 1.12 | | | | | | 0.41 | | | | | 3.05 | N/D^1^ | | | | | |
| rs1652508 | N/A | | | | | | | | | | | | | | | | | | | | | | | | | | | |
| rs1830521 | 0.62 | | | | 0.79 | | | | | | 1.15 | | | | | | 0.67 | | | | | 1.98 | 0.08 | | 0.20 | 0.56 | 0.29 | 1.07 |
| rs1835346 | 0.92 | | | | 0.97 | | | | | | 1.07 | | | | | | 0.29 | | | | | 3.89 | N/D^1,2^ | | | | | |
| rs2144723 | 0.29 | | | | 0.51 | | | | | | 0.73 | | | | | | 0.41 | | | | | 1.31 | 0.30 | | 0.52 | 1.41 | 0.74 | 2.68 |
| rs4252050 | 0.80 | | | | 0.91 | | | | | | 1.07 | | | | | | 0.63 | | | | | 1.82 | 0.31 | | 0.53 | 0.46 | 0.10 | 2.08 |
| rs4252053 | 0.02 | | | | 0.08 | | | | | | 1.97 | | | | | | 1.10 | | | | | 3.54 | 0.79 | | 0.91 | 1.26 | 0.23 | 7.00 |
| rs4252055 | 0.52 | | | | 0.73 | | | | | | 0.77 | | | | | | 0.36 | | | | | 1.68 | N/D^1,2^ | | | | | |
| rs4252064 | N/A | | | | | | | | | | | | | | | | | | | | | | | | | | | |
| rs4252069 | N/A | | | | | | | | | | | | | | | | | | | | | | | | | | | |
| rs4252075 | N/A | | | | | | | | | | | | | | | | | | | | | | | | | | | |
| rs4252087 | N/A | | | | | | | | | | | | | | | | | | | | | | | | | | | |
| rs4252099 | N/A | | | | | | | | | | | | | | | | | | | | | | | | | | | |
| rs4252108 | N/A | | | | | | | | | | | | | | | | | | | | | | | | | | | |
| rs4252125 | 0.82 | | | | 0.92 | | | | | | 0.94 | | | | | | 0.57 | | | | | 1.58 | 0.78 | | 0.90 | 1.16 | 0.41 | 3.24 |
| rs4252159 | 0.82 | | | | 0.92 | | | | | | 0.91 | | | | | | 0.41 | | | | | 2.02 | N/D^1,2^ | | | | | |
| rs4252166 | 0.36 | | | | 0.58 | | | | | | 0.77 | | | | | | 0.44 | | | | | 1.34 | 0.43 | | 0.65 | 1.92 | 0.38 | 9.74 |
| rs4252181 | N/A | | | | | | | | | | | | | | | | | | | | | | | | | | | |
| rs4252185 | 0.93 | | | | 0.98 | | | | | | 0.97 | | | | | | 0.49 | | | | | 1.92 | 0.74 | | 0.87 | 0.63 | 0.04 | 10.17 |
| rs4252192 | 0.63 | | | | 0.80 | | | | | | 0.79 | | | | | | 0.31 | | | | | 2.04 | N/D^1,2^ | | | | | |
| rs4252200 | 0.55 | | | | 0.75 | | | | | | 0.77 | | | | | | 0.33 | | | | | 1.80 | N/D^1^ | | | | | |
| rs4709458 | 0.14 | | | | | 0.32 | | | | | | 1.50 | | | | | | 0.87 | | | | 2.59 | 0.52 | | 0.73 | 0.78 | 0.37 | 1.66 |
| rs7454268 | 0.18 | | | | | 0.38 | | | | | | 1.43 | | | | | | 0.84 | | | | 2.42 | 0.35 | | 0.57 | 0.65 | 0.27 | 1.61 |
| rs783146 | 0.98 | | | | | 1.00 | | | | | | 0.99 | | | | | | 0.55 | | | | 1.77 | 0.40 | | 0.63 | 2.56 | 0.28 | 23.24 |
| rs783147 | 0.86 | | | | | 0.95 | | | | | | 1.05 | | | | | | 0.60 | | | | 1.83 | 0.22 | | 0.42 | 1.54 | 0.78 | 3.07 |
| rs783167 | N/A | | | | | | | | | | | | | | | | | | | | | | | | | | | |
| rs783176 | 0.63 | | | | | 0.80 | | | | | | 0.87 | | | | | | 0.49 | | | | 1.53 | 0.41 | | 0.63 | 2.54 | 0.28 | 23.08 |
| rs783182 | N/A | | | | | | | | | | | | | | | | | | | | | | | | | | | |
| rs9295131 | 0.33 | | | | | 0.55 | | | | | | 0.77 | | | | | | 0.46 | | | 1.30 | | 0.73 | | 0.87 | 1.18 | 0.45 | 3.08 |
| rs9456577 | 0.17 | | | | | 0.36 | | | | | | 2.49 | | | | | | 0.68 | | | 9.06 | | N/D^1,2^ | | | | | |
| rs9458011 | 0.86 | | | | | 0.94 | | | | | | 0.93 | | | | | | 0.43 | | | 2.01 | | N/D^1,2^ | | | | | |
| **PTX3** |  | | | | |  | | | | | |  | | | | | |  | | |  | |  | |  |  |  |  |
| rs1840680 | 0.41 | | | | | 0.63 | | | | | | 1.29 | | | | | | 0.70 | | | 2.39 | | 0.41 | | 0.63 | 0.78 | 0.43 | 1.41 |
| rs3845978 | 0.44 | | | | | 0.66 | | | | | | 1.40 | | | | | | 0.60 | | | 3.27 | | N/D^1,2^ | | | | | |
| rs3911403 | 0.82 | | | | | 0.92 | | | | | | 0.91 | | | | | | 0.42 | | | 1.98 | | N/D^1,2^ | | | | | |
| **STAT3** |  | | | | |  | | | | | |  | | | | | |  | | |  | |  | |  |  |  |  |
| rs1026916 | 0.26 | | | | | 0.47 | | | | | | 0.74 | | | | | | 0.43 | | | 1.25 | | 0.93 | | 0.98 | 1.03 | 0.50 | 2.12 |
| rs12103893 | 0.94 | | | | | 0.98 | | | | | | 0.98 | | | | | | 0.57 | | | 1.68 | | 0.98 | | 1.00 | 1.02 | 0.28 | 3.70 |
| rs17880347 | 0.57 | | | | | 0.76 | | | | | | 0.80 | | | | | | 0.38 | | | 1.70 | | N/D^1^ | | | | | |
| rs17880900 | N/A | | | | | | | | | | | | | | | | | | | | | | | | | | | |
| rs17881320 | N/A | | | | | | | | | | | | | | | | | | | | | | | | | | | |
| rs17881940 | 0.22 | | | | | 0.43 | | | | | | 0.63 | | | | | | 0.30 | | | 1.32 | | 0.70 | | 0.85 | 0.68 | 0.09 | 4.90 |
| rs1905340 | 0.20 | | | | | 0.41 | | | | | | 0.72 | | | | | | 0.43 | | | 1.20 | | 0.90 | | 0.97 | 0.95 | 0.42 | 2.13 |
| rs2293152 | 0.36 | | | | | 0.57 | | | | | | 1.28 | | | | | | 0.76 | | | 2.18 | | 0.28 | | 0.50 | 0.66 | 0.31 | 1.40 |
| rs2306580 | 0.51 | | | | | 0.73 | | | | | | 1.29 | | | | | | 0.61 | | | 2.73 | | N/D^1^ | | | | | |
| rs2306581 | N/A | | | | | | | | | | | | | | | | | | | | | | | | | | | |
| rs6503695 | 0.26 | | | | | 0.47 | | | | | | 0.74 | | | | | | 0.43 | | | 1.25 | | 0.76 | | 0.89 | 1.12 | 0.53 | 2.37 |
| rs6503697 | N/A | | | | | | | | | | | | | | | | | | | | | | | | | | | |
| rs7217655 | N/A | | | | | | | | | | | | | | | | | | | | | | | | | | | |
| rs744166 | 0.28 | | | | | 0.50 | | | | | | 0.74 | | | | | | 0.42 | | | 1.28 | | 0.44 | | 0.66 | 1.30 | 0.67 | 2.53 |
| rs8075442 | N/D^3^ | | | | | | | | | | | | | | | | | | | | | | N/D^1^ | | | | | |
| rs8078731 | N/A | | | | | | | | | | | | | | | | | | | | | | | | | | | |
| rs957971 | N/A | | | | | | | | | | | | | | | | | | | | | | | | | | | |
| **TLR1** |  | | | | |  | | | | | |  | | | | | |  | | |  | |  | |  |  |  |  |
| rs4540055 | N/A | | | | | | | | | | | | | | | | | | | | | | | | | | | |
| rs4833095 | 0.68 | | | | | 0.84 | | | | | | 0.89 | | | | | | 0.50 | | | 1.56 | | 0.49 | | 0.71 | 0.66 | 0.21 | 2.12 |
| rs5743551 | N/A | | | | | | | | | | | | | | | | | | | | | | | | | | | |
| rs5743565 | N/A | | | | | | | | | | | | | | | | | | | | | | | | | | | |
| rs5743594 | 0.42 | | | | | 0.64 | | | | | | 1.24 | | | | | | 0.73 | | | 2.12 | | 0.99 | | 1.00 | 1.01 | 0.28 | 3.68 |
| rs5743595 | 0.82 | | | | | 0.92 | | | | | | 1.07 | | | | | | 0.59 | | | 1.94 | | 0.89 | | 0.97 | 0.90 | 0.20 | 4.12 |
| rs5743611 | 0.30 | | | | | 0.52 | | | | | | 1.49 | | | | | | 0.71 | | | 3.14 | | N/D^2^ | | | | | |
| rs5743618 | N/A | | | | | | | | | | | | | | | | | | | | | | | | | | | |
| **TLR2** |  | | | | |  | | | | | |  | | | | | |  | | |  | |  | |  |  |  |  |
| rs1816702 | 0.78 | | | | | 0.90 | | | | | | 0.91 | | | | | | 0.48 | | | 1.74 | | 0.58 | | 0.76 | 1.89 | 0.19 | 18.46 |
| rs1898830 | 0.34 | | | | | 0.56 | | | | | | 1.28 | | | | | | 0.77 | | | 2.15 | | 0.64 | | 0.80 | 1.21 | 0.54 | 2.74 |
| rs3804099 | 0.91 | | | | | 0.97 | | | | | | 0.97 | | | | | | 0.57 | | | 1.66 | | 0.72 | | 0.86 | 0.89 | 0.48 | 1.65 |
| rs3804100 | 0.15 | | | | | 0.34 | | | | | | 1.72 | | | | | | 0.82 | | | 3.63 | | N/D^1^ | | | | | |
| rs4696480 | 0.42 | | | | | 0.64 | | | | | | 1.28 | | | | | | 0.71 | | | 2.29 | | 0.84 | | 0.93 | 1.07 | 0.56 | 2.02 |
| rs5743704 | 0.54 | | | | | 0.74 | | | | | | 0.78 | | | | | | 0.35 | | | 1.74 | | N/D^1^ | | | | | |
| rs5743708 | 0.15 | | | | | 0.33 | | | | | | 0.43 | | | | | | 0.14 | | | 1.36 | | N/D^1,2^ | | | | | |
| rs7656411 | 0.85 | | | | | 0.94 | | | | | | 1.05 | | | | | | 0.63 | | | 1.77 | | 0.36 | | 0.57 | 0.66 | 0.27 | 1.61 |
| **TLR3** |  | | | | |  | | | | | |  | | | | | |  | | |  | |  | |  |  |  |  |
| rs10025405 | 0.03 | | | | | 0.10 | | | | | | 1.83 | | | | | | 1.05 | | | 3.18 | | 0.86 | | 0.94 | 0.94 | 0.49 | 1.80 |
| rs11721827 | 0.34 | | | | | 0.56 | | | | | | 1.32 | | | | | | 0.75 | | | 2.33 | | 0.43 | | 0.65 | 1.91 | 0.38 | 9.67 |
| rs1879026 | 0.01 | | | | | 0.03 | | | | | | 0.44 | | | | | | 0.24 | | | 0.80 | | N/D^1^ | | | | | |
| rs3775291 | 0.43 | | | | | 0.65 | | | | | | 0.81 | | | | | | 0.49 | | | 1.36 | | 0.70 | | 0.85 | 1.19 | 0.48 | 2.93 |
| rs3775292 | 0.54 | | | | | 0.74 | | | | | | 0.85 | | | | | | 0.50 | | | 1.44 | | 0.79 | | 0.90 | 1.27 | 0.23 | 7.05 |
| rs5743303 | 0.02 | | | | | 0.06 | | | | | | 1.95 | | | | | | 1.13 | | | 3.36 | | 0.82 | | 0.92 | 0.87 | 0.27 | 2.82 |
| rs5743305 | 0.02 | | | | | 0.07 | | | | | | 0.54 | | | | | | 0.32 | | | 0.91 | | 0.61 | | 0.79 | 1.22 | 0.56 | 2.67 |
| rs5743313 | N/A | | | | | | | | | | | | | | | | | | | | | | | | | | | |
| rs5743314 | N/A | | | | | | | | | | | | | | | | | | | | | | | | | | | |
| rs6552950 | 0.93 | | | | | 0.98 | | | | | | 0.98 | | | | | | 0.57 | | | 1.66 | | 0.57 | | 0.76 | 0.77 | 0.32 | 1.87 |
| rs7657186 | 0.63 | | | | | 0.80 | | | | | | 0.88 | | | | | | 0.51 | | | 1.50 | | 0.74 | | 0.87 | 0.83 | 0.28 | 2.47 |
| rs7668666 | 0.04 | | | | | 0.10 | | | | | | 1.75 | | | | | | 1.04 | | | 2.96 | | 0.33 | | 0.55 | 1.62 | 0.61 | 4.34 |
| **TLR4** |  | | | | |  | | | | | |  | | | | | |  | | |  | |  | |  |  |  |  |
| rs10759931 | 0.65 | | | | | 0.82 | | | | | | 0.89 | | | | | | 0.53 | | | 1.49 | | 0.50 | | 0.72 | 1.33 | 0.58 | 3.05 |
| rs11536857 | 0.68 | | | | | 0.84 | | | | | | 1.14 | | | | | | 0.61 | | | 2.15 | | 0.70 | | 0.85 | 0.58 | 0.04 | 9.33 |
| rs11536869 | 0.48 | | | | | | 0.70 | | | | | | 1.42 | | | | | | 0.54 | | 3.72 | | N/D^2^ | | | | | |
| rs11536878 | 0.16 | | | | | | 0.34 | | | | | | 0.63 | | | | | | 0.33 | | 1.20 | | 0.63 | | 0.80 | 1.75 | 0.18 | 17.08 |
| rs11536889 | 0.59 | | | | | | 0.77 | | | | | | 1.16 | | | | | | 0.67 | | 2.01 | | 0.30 | | 0.52 | 0.28 | 0.03 | 3.14 |
| rs11536891 | 0.78 | | | | | | 0.90 | | | | | | 1.08 | | | | | | 0.62 | | 1.90 | | 0.83 | | 0.93 | 1.16 | 0.28 | 4.77 |
| rs11536897 | 0.87 | | | | | | 0.95 | | | | | | 0.92 | | | | | | 0.36 | | 2.40 | | N/D^1,2^ | | | | | |
| rs12377632 | N/A | | | | | | | | | | | | | | | | | | | | | | | | | | | |
| rs1554973 | 0.40 | | | | | | 0.63 | | | | | | 1.24 | | | | | | 0.75 | | 2.06 | | 0.38 | | 0.60 | 0.70 | 0.31 | 1.56 |
| rs1927906 | 0.19 | | | | | | 0.40 | | | | | | 1.46 | | | | | | 0.83 | | 2.57 | | 0.70 | | 0.85 | 0.58 | 0.04 | 9.33 |
| rs1927907 | 0.07 | | | | | | 0.18 | | | | | | 1.69 | | | | | | 0.96 | | 2.96 | | 0.47 | | 0.70 | 0.55 | 0.11 | 2.80 |
| rs2149356 | 0.30 | | | | | | 0.52 | | | | | | 1.31 | | | | | | 0.78 | | 2.20 | | 0.56 | | 0.76 | 0.80 | 0.39 | 1.68 |
| rs4986791 | 0.21 | | | | | | 0.42 | | | | | | 1.51 | | | | | | 0.79 | | 2.86 | | N/D^1^ | | | | | |
| rs5030728 | 0.38 | | | | | | 0.61 | | | | | | 0.80 | | | | | | 0.48 | | 1.33 | | 0.84 | | 0.93 | 0.92 | 0.40 | 2.12 |
| rs4986790 | N/A | | | | | | | | | | | | | | | | | | | | | | | | | | | |
| **TLR9** |  | | | | | |  | | | | | |  | | | | | |  | |  | |  | |  |  |  |  |
| rs187084 | 0.77 | | | | | | 0.89 | | | | | | 0.92 | | | | | | 0.54 | | 1.58 | | 0.38 | | 0.60 | 0.74 | 0.37 | 1.46 |
| rs352140 | 0.59 | | | | | | 0.77 | | | | | | 0.86 | | | | | | 0.50 | | 1.48 | | 0.58 | | 0.76 | 0.84 | 0.45 | 1.58 |
| rs5743836 | 0.92 | | | | | | 0.97 | | | | | | 0.97 | | | | | | 0.56 | | 1.70 | | 0.58 | | 0.76 | 1.59 | 0.30 | 8.38 |
| **TLR10** |  | | | | | |  | | | | | |  | | | | | |  | |  | |  | |  |  |  |  |
| rs10856838 | 0.07 | | | | | | 0.18 | | | | | | 1.76 | | | | | | 0.96 | | 3.22 | | N/D^1^ | | | | | |
| rs11096955 | 0.83 | | | | | | 0.93 | | | | | | 1.06 | | | | | | 0.63 | | 1.77 | | 0.87 | | 0.95 | 1.09 | 0.41 | 2.86 |
| rs11466640 | 0.28 | | | | | | 0.50 | | | | | | 0.72 | | | | | | 0.39 | | 1.31 | | 0.95 | | 0.99 | 1.04 | 0.24 | 4.47 |
| rs11466652 | 0.10 | | | | | | 0.24 | | | | | | 1.67 | | | | | | 0.91 | | 3.07 | | N/D^1^ | | | | | |
| rs11466657 | N/A | | | | | | | | | | | | | | | | | | | | | | | | | | | |
| rs4129009 | 0.28 | | | | | | 0.50 | | | | | | 0.72 | | | | | | 0.39 | | 1.31 | | 0.95 | | 0.99 | 1.04 | 0.24 | 4.47 |
| rs4513579 | N/A | | | | | | | | | | | | | | | | | | | | | | | | | | | |
| rs7658893 | 0.70 | | | | | | 0.85 | | | | | | 0.90 | | | | | | 0.51 | | 1.57 | | 0.99 | | 1.00 | 1.01 | 0.32 | 3.17 |
| **TREM1** |  | | | | | |  | | | | | |  | | | | | |  | |  | |  | |  |  |  |  |
| rs12200981 | 0.77 | | | | | | 0.89 | | | | | | 1.09 | | | | | | 0.61 | | 1.96 | | 0.32 | | 0.54 | 1.33 | 0.75 | 2.35 |
| rs1351835 | N/A | | | | | | | | | | | | | | | | | | | | | | | | | | | |
| rs2234237 | N/D^4,5^ | | | | | | | | | | | | | | | | | | | | | | N/D^1,2^ | | | | | |
| rs2234243 | 0.92 | | | | | | 0.97 | | | | | | 0.97 | | | | | | 0.50 | | 1.88 | | 0.58 | | 0.76 | 1.89 | 0.19 | 18.46 |
| rs3789204 | 0.05 | | | | | | 0.15 | | | | | | 1.66 | | | | | | 0.99 | | 2.79 | | 0.33 | | 0.54 | 1.64 | 0.61 | 4.38 |
| rs3827632 | N/A | | | | | | | | | | | | | | | | | | | | | | | | | | | |
| rs4711668 | 0.97 | | | | | | 1.00 | | | | | | 1.01 | | | | | | 0.61 | | 1.69 | | 0.31 | | 0.53 | 1.53 | 0.67 | 3.50 |
| rs6939973 | 0.90 | | | | | | 0.97 | | | | | | 1.04 | | | | | | 0.60 | | 1.78 | | N/D^1^ | | | | | |
| rs6940092 | 0.52 | | | | | | 0.73 | | | | | | 1.19 | | | | | | 0.70 | | 2.03 | | 0.21 | | 0.42 | 1.54 | 0.79 | 3.00 |

All SNPs are shown. N/A, SNPs that were not analysed (see Details of method of generating MDMs for details). N/D, model not done as no subjects within one of the comparison groups (details given by superscript). ^1^, No minor homozygotes in ABPA group. ^2^, No minor homozygotes in Asthma group. ^3^, all major homozygotes in the ABPA (no heterozygous, no minor homozygous). ^4^, No major homozygotes in ABPA group. ^5^, No major homozygotes in Asthma group

Supplementary Table 3 - Genotype frequencies for the ABPA and healthy groups, for SNPs associated with ABPA in the ABPA vs. atopic asthma comparison

| Gene | SNP | Alleles (M/m) | Model for association |  | Genotype Freq ABPA vs. Healthy | | |
| --- | --- | --- | --- | --- | --- | --- | --- |
|  |  |  |  | Genotype | ABPA | Healthy | p-value |
| **ADORA2A** | rs2236624 | C/**T** | CC+CT vs. TT | TT  CC+CT | 11 (11.5%)  84 (88.4%) | 18 (6.5%)  261 (93.5%) | 0.107 |
| **DECTIN1** | rs11053624 | T/**C** | CC+TC vs. TT | TT  CC+CT | 72 (75.8%)  23 (24.2%) | 233 (83.5%)  46 (16.5%) | 0.094 |
|  | rs7959451 | C/**T** | TT+CT vs. CC | CC  TT+CT | 62 (65.3%)  33 (34.7%) | 212 (76.0%)  67 (24.0%) | **0.042** |
| **IL13** | rs20541 | G/**A** | AA+GA vs. GG | GG  AA+GA | 51 (53.7%)  44 (46.3%) | 167 (61.4%)  105 (38.6%) | 0.188 |
|  | rs1800925 | C/**T** | TT+TC vs. CC | CC  TT+TC | 53 (55.8%)  42 (44.2%) | 186 (68.1%)  87 (31.9%) | **0.030** |
| **IL17A** | rs3819024 | A/**G** | GG+GA vs. AA | AA  GG+GA | 33 (34.7%)  62 (65.3%) | 115 (41.2%)  164 (58.8%) | 0.265 |
| **IL4R** | rs3024656 | **G**/A | GG+GA vs. AA | AA  GG+GA | 3 (3.2%)  92 (96.8%) | 33 (12.1%)  239 (87.9%) | **0.012** |
|  | rs1029489 | G/**A** | AA+GA vs. GG | GG  AA+GA | 24 (25.3%)  71 (74.7%) | 113 (41.4%)  160 (58.6%) | **0.003** |
|  | rs6498012 | G/C | GG+GC vs. CC | CC  GG+GC | 20 (21.1%)  75 (78.9%) | 35 (12.9%)  237 (87.1%) | 0.055 |
| **MBL2** | rs2099903 | C/**A** | CC+CA vs. AA | AA  CC+CA | 11 (11.6%)  84 (88.4%) | 21 (7.5%)  258 (92.5%) | 0.223 |
| **PLAT** | rs8178880 | **A**/G | GG+AG vs. AA | AA  GG+AG | 91 (96.8%)  3 (3.2%) | 256 (91.8%)  23 (8.2%) | 0.097 |
| **PLG** | rs4252053 | A/**G** | GG+AG vs. AA | AA  GG+AG | 64 (67.4%)  31 (32.6%) | 194 (69.5%)  85 (30.5%) | 0.694 |
| **TLR3** | rs1879026 | **G**/T | TT+GT vs. GG | GG  TT+GT | 75 (78.9%)  20 (21.1%) | 197 (70.6%)  82 (29.4%) | 0.115 |
|  | rs10025405 | A/**G** | GG+GA vs. AA | AA  GG+GA | 26 (27.4%)  69 (72.6%) | 95 (34.1%)  184 (65.9%) | 0.230 |
|  | rs5743303 | A/**T** | TT+AT vs. AA | AA  TT+AT | 56 (58.9%)  39 (41.1%) | 184 (65.9%)  95 (34.1%) | 0.220 |
|  | rs5743305 | **T**/A | AA+TA vs. TT | TT  AA+TA | 48 (50.5%)  47 (49.5%) | 117 (41.9%)  162 (58.1%) | 0.146 |
|  | rs7668666 | C/**A** | AA+CA vs. CC | CC  AA+CA | 49 (51.6%)  46 (48.4%) | 164 (58.8%)  115 (41.2%) | 0.221 |

Risk allele from the ABPA v Atopic asthma comparison is shown in bold. p-value calculated for the model indicated using chi-squared tests in SVS (for ABPA v Healthy). Significant p-values (p<0.05) shown in bold. CI, Confidence interval. M/m, Major allele/Minor allele.

Supplementary Figure 1 - Expression of TLR downstream signalling molecules by MDMs from ABPA, asthmatic and healthy subjects (n=10, pooled). Panels A-C show expression in the ABPA (black bars) and asthma groups (grey bars) relative to the healthy group (dotted line) at each timepoint. Stars indicate significant differences between ABPA and asthma, calculated by t-test. Panels D-H show expression in ABPA (open circles), asthmatic (closed squares) and healthy (closed triangles) subjects over time, relative to asthma 0hr. Stars indicate significant changes over time, calculated by repeated measures 1-way ANOVA. Bars indicate standard deviation. *, p<0.05, **, p<0.01; ***, p<0.001; ****, p<0.0001.

Supplementary Figure 2 - Additional expression by MDMs from ABPA, asthmatic and healthy subjects (n=10, pooled). Panels A-B show expression in the ABPA (black bars) and asthma groups (grey bars) relative to the healthy group (dotted line) at each timepoint. Stars indicate significant differences between ABPA and asthma, calculated by t-test. Panels C-F show expression in ABPA (open circles), asthmatic (closed squares) and healthy (closed triangles) subjects over time, relative to asthma 0hr. Stars indicate significant changes over time, calculated by repeated measures 1-way ANOVA. Bars indicate standard deviation. *, p<0.05, **, p<0.01; ***, p<0.001; ****, p<0.0001.

1. **Loeffler J, Haddad Z, Bonin M, Romeike N, Mezger M, Schumacher U, Kapp M, Gebhardt F, Grigoleit GU, Stevanovic S, Einsele H, Hebart H.** 2009. Interaction analyses of human monocytes co-cultured with different forms of Aspergillus fumigatus. J. Med. Microbiol. **58:**49-58.

2. **Smith NL, Hankinson J, Simpson A, Bowyer P, Denning DW.** 2014. A prominent role for the IL1 pathway and IL15 in susceptibility to chronic cavitary pulmonary aspergillosis. Clin Microbiol Infect **20:**O480-488.
